# Metabolomic and transcriptomic profiling of adult mice and larval zebrafish leptin mutants reveal a common pattern of changes in metabolites and signaling pathways

**DOI:** 10.1186/s13578-021-00642-0

**Published:** 2021-07-07

**Authors:** Yi Ding, Mariëlle C. Haks, Gabriel Forn-Cuní, Junling He, Natalia Nowik, Amy C. Harms, Thomas Hankemeier, Muhamed N. H. Eeza, Jörg Matysik, A. Alia, Herman P. Spaink

**Affiliations:** 1 Institute of Biology, Leiden University, Sylviusweg 72, 2333 BE Leiden, The Netherlands; 2 Department of Infectious Diseases, Leiden University Medical Center, Leiden, The Netherlands; 3 Department of Animal Anatomy, Faculty of Veterinary Medicine, University of Warmia and Mazury, Oczapowskiego 13, 10-719 Olsztyn, Poland; 4 Leiden Academic Centre for Drug Research, Leiden University, Leiden, The Netherlands; 5 Institute of Medical Physics and Biophysics, University of Leipzig, 04107 Leipzig, Germany; 6 Institute of Analytical Chemistry, University of Leipzig, Linnéstraße 3, 04103 Leipzig, Germany; 7 Leiden Institute of Chemistry, Leiden University, Leiden, The Netherlands

**Keywords:** *Ob/ob* mice, *Leptin* mutant zebrafish, Diabetes, Metabolomics, Transcriptomics, Wasting syndrome

## Abstract

**Background:**

Leptin plays a critical role in the regulation of metabolic homeostasis. However, the molecular mechanism and cross talks between leptin and metabolic pathways leading to metabolic homeostasis across different species are not clear. This study aims to explore the effects of leptin in mice and zebrafish larvae by integration of metabolomics and transcriptomics. Different metabolomic approaches including mass spectrometry, nuclear magnetic resonance (NMR) and high-resolution magic-angle-spinning NMR spectrometry were used to investigate the metabolic changes caused by *leptin* deficiency in mutant *ob/ob* adult mice and *lepb*^*−/−*^ zebrafish larvae. For transcriptome studies, deep RNA sequencing was used.

**Results:**

Thirteen metabolites were identified as common biomarkers discriminating *ob/ob* mice and *lepb*^−/−^ zebrafish larvae from their respective wild type controls: alanine, citrulline, ethanolamine, glutamine, glycine, histidine, isoleucine, leucine, methionine, phenylalanine, putrescine, serine and threonine. Moreover, we also observed that glucose and lipid levels were increased in *lepb*^−/−^ zebrafish larvae compared to the *lepb*^+/+^ group. Deep sequencing showed that many genes involved in proteolysis and arachidonic acid metabolism were dysregulated in *ob/ob* mice heads and *lepb* mutant zebrafish larvae compared to their wild type controls, respectively.

**Conclusions:**

*Leptin* deficiency leads to highly similar metabolic alterations in metabolites in both mice and zebrafish larvae. These metabolic changes show similar features as observed during progression of tuberculosis in human patients, mice and zebrafish larvae. In addition, by studying the transcriptome, we found similar changes in gene regulation related to proteolysis and arachidonic acid metabolism in these two different in vivo models.

**Supplementary Information:**

The online version contains supplementary material available at 10.1186/s13578-021-00642-0.

## Background

Leptin, the first discovered adipokine, plays a critical role in the regulation of energy balance and homeostasis of metabolism [1, 2]. Congenital leptin deficiency in humans results in extreme obesity, hyperphagia and many complications such as type 2 diabetes [3]. Leptin administration therapy with metreleptin, a recombinant human leptin analogue, has been approved for the treatment of the metabolic abnormalities linked to dyslipidemia [4]. Metabolic effects of leptin have been studied in rodent animal models [5]. Leptin signaling deficient rodent mutants, such as *ob/ob* mice, *db/db* mice and Zucker rats, have been commonly used as animal models in leptin studies [5]. Similar to the rare cases of congenital human leptin deficiency, these rodent mutants display hyperphagia, obesity and an insulin resistant phenotype. Several studies have shown metabolic disorders in *ob/ob* mice [6–8], *db/db* mice [6, 9, 10] and obese Zucker rats [11] measured by mass spectrometry (MS) or ^1^H solution nuclear magnetic resonance (NMR). Using a positional isotopomer NMR tracer analysis method, Perry et al. showed that leptin mediates a glucose-fatty acid cycle to maintain glucose homeostasis in starvation in rats [12]. Using a combination of metabolomics and transcriptomics, a recent published paper demonstrates that the carbohydrate, lipid and amino acid metabolic liver responses to glucose administration are broadly different between wild type and *ob/ob* mice [13].

Leptin and leptin receptor (*lepr*) are highly conserved and share extensive homology across vertebrates including all mammals and fish and have been studied in many model organisms [14, 15]. However, leptin functions in early development of vertebrates are largely unknown. Notwithstanding many reports indicate that leptin plays a key role in gestational diabetes and fetal development [16–20]. Further understanding of the function of leptin in these processes is hampered by the challenges of using rodent animal models for the study of fetal development. Zebrafish represents a robust animal model for early development because of its external fertilization, transparency of its larvae and large numbers of offspring. Since the zebrafish larvae are independent of feeding in the first five days after fertilization, it offers a great model for comparative leptin studies in fetal development with adult mammals. In zebrafish, there are two orthologs of the human leptin gene, *leptin a* (*lepa*) and *leptin b* (*lepb*). A zebrafish mutant line with *lepa* gene deficiency displays a phenotype of obesity and various deviations in behavior and circadian rhythm in the adult stage [21]. It was shown that *lepb* mutant zebrafish have more visceral fat and higher glucose level in male adult fish [22]. However, a zebrafish mutant for *lepr* was reported not to exhibit increased obesity in adult fish [23]. In larval studies, we have previously shown that *lepb* is one of the most affected genes after insulin injection [24]. However, how *lepb* gene affects the metabolic and transcriptomic level in zebrafish larvae is still unknown.

In this study, we have compared the metabolic changes resulting from *leptin* deficiency in blood of adult *ob/ob* mice and extracted and intact zebrafish larvae using MS, solution-state NMR and high-resolution magic-angle-spinning NMR (HR-MAS NMR) spectrometry. HR-MAS NMR is a noninvasive method that can be used for analysis of intact tissues at low temperature. In addition, we have compared the transcriptomic changes resulting from *leptin* deficiency in *ob/ob* mice heads, a published dataset for *ob/ob* mice liver and *lepb* mutant zebrafish larvae. These comparisons show a remarkable similarity of the effects of *leptin* knockdown on the metabolomes and transcriptomes of adult mice and zebrafish larvae.

## Results

### Metabolic profiles of blood from *ob/ob* and wild type mice measured by MS

**Fig. 1:**
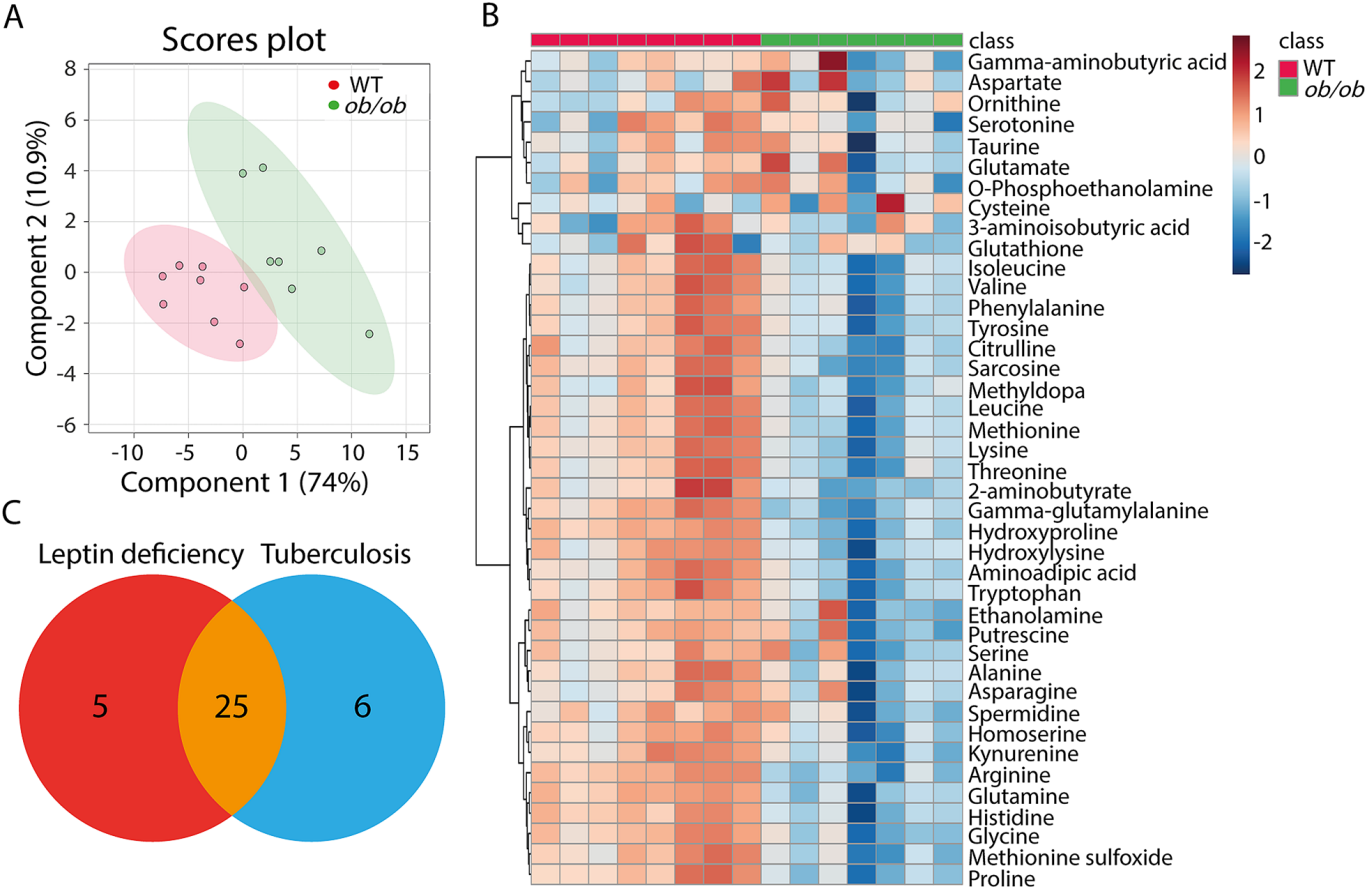
Metabolic profiles of blood from *ob/ob* and wild type C57BL/6 mice measured by mass spectrometry. **A** PLS-DA analysis of wild type and *ob/ob* mice, n = 15 in total, each replicate represents one mouse. *PLS-DA* partial least square discriminant analysis. *WT* wild type. **B** Heat map of 30 statistically significant biochemical markers profiled in this mice study. **C** A Venn diagram showing the overlap of the 30 metabolites of **B** with the set of wasting syndrome biomarkers published by Ding et al. [26]

We first investigated the metabolic profiles of blood from *ob/ob* and wild type lean male mice at 14 weeks of age. Mice were kept on a standard diet for 8 weeks, after which the body weight of *ob/ob* mice was significantly higher than wild type C57BL/6 mice (Additional file 1: Figure S1). Metabolic profiles of the blood of the two groups were obtained by MS. Using a highly standardized platform we could measure 41 small amine-containing compounds. A Partial Least Squares Discriminant Analysis (PLS-DA) scores plot of the 41 identified metabolites showed clear differences between the *ob/ob* and the wild type mice, indicating metabolic alterations in the metabolism due to *leptin* deficiency (Fig. 1A). Using a cut-off *p* value of 0.05, we could classify 30 out of the 41 identified small amine-containing compounds as associated with *ob/ob* mice. These 30 metabolites were significantly downregulated with a *p* value < 0.05 in *ob/ob* mice compared to wild type mice (Fig. 1B, Additional file 1: Table S1). For 25 of these metabolites, we have previously shown that they are biomarkers for *Mycobacterium tuberculosis* (*Mtb*)-infected mice (Fig. 1C). Graphs showing the quantification of these 25 common metabolites revealed that both the original and normalized values were decreased in *leptin*-deficient *ob/ob* mice (Additional file 1: Figure S2).

### Metabolic profiles of extracts of *lepb* deficient and wild type zebrafish larvae measured by NMR

**Fig. 2:**
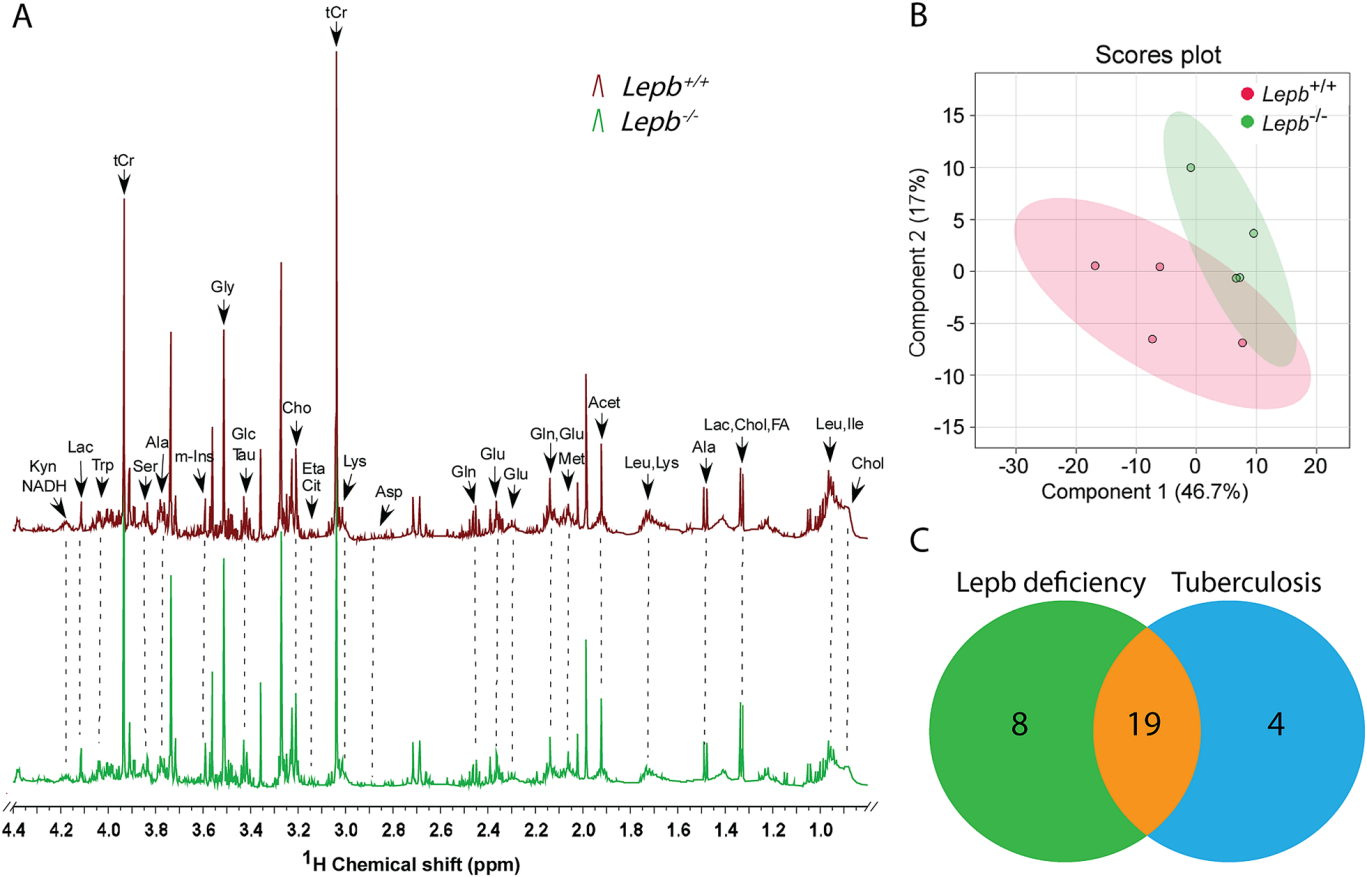
One-dimensional ^1^H NMR spectra and PLS-DA analysis of extracted *lepb* mutant zebrafish larvae. **A** The representative spectra of extracted larvae from wild type and *lepb* mutant groups measured by solution NMR spectrometry. Spectra from chemical shift 0.5–4.4 were assigned to specific metabolites. *Acet* acetate, *Ala* alanine, *Arg* arginine, *Asp* aspartate, *Cho* choline, *Chol* cholesterol, *Cit* citrulline, *Eta* ethanolamine, *FA* fatty acid, *Glc* glucose, *Gln* glutamine, *Glu* glutamate, *Gly* glycine, Ile isoleucine, *Kyn* kynurenine, *Lac* lactate, *Leu* leucine, *Lys* lysine, *Met* methionine, *m-Ins* myo-inositol, *Ser* serine, *Tau* taurine, *tCr* total creatine (creatine + phosphocreatine), *Trp* tryptophan, *NMR* nuclear magnetic resonance. **B** PLS-DA analysis of wild type and *lepb* mutant groups, n = 4, each replicate represents 105 pooled larvae. *PLS-DA* partial least square discriminant analysis. **C** A Venn diagram is shown of the common 19 metabolites that changed significantly towards *lepb* deficiency in extracted zebrafish larvae and tuberculosis caused by *Mycobacterium marinum* infection in extracted zebrafish larvae published by Ding et al. [26]

A *lepb* mutant zebrafish line was generated by CRISPR/CAS methodology [22]. Metabolic profiles of extracted zebrafish larvae from *lepb*^−/−^ mutant and *lepb*^+/+^ wild type siblings were measured by one-dimensional ^1^H solution NMR. Figure 2A shows the representative ^1^H NMR spectra of extracted metabolites in the two groups. The assignment was performed based on the peaks of reference metabolites from literature [6, 25] in the library of Chenomx 600 MHz (version 11). A PLS-DA scores plot showed differences between the *lepb*^−/−^ and *lepb*^+/+^ groups (Fig. 2B), suggesting metabolic changes resulting from *lepb* deficiency in zebrafish larvae. We found 27 metabolites to be significantly changed with a *p* value < 0.05 in extracted *lepb*^−/−^ zebrafish larvae compared to *lepb*^+/+^ group. For 19 of these metabolites, we have previously shown that they are biomarkers for *Mycobacterium marinum*-infected zebrafish larvae (Fig. 2C). Quantification of these 19 common metabolites showed that the levels of all the metabolites were decreased in *lepb* mutant zebrafish larvae (Additional file 1: Figure S3).

### Metabolic profiles of intact *lepb* deficient and wild type zebrafish larvae measured by HR-MAS NMR

**Fig. 3:**
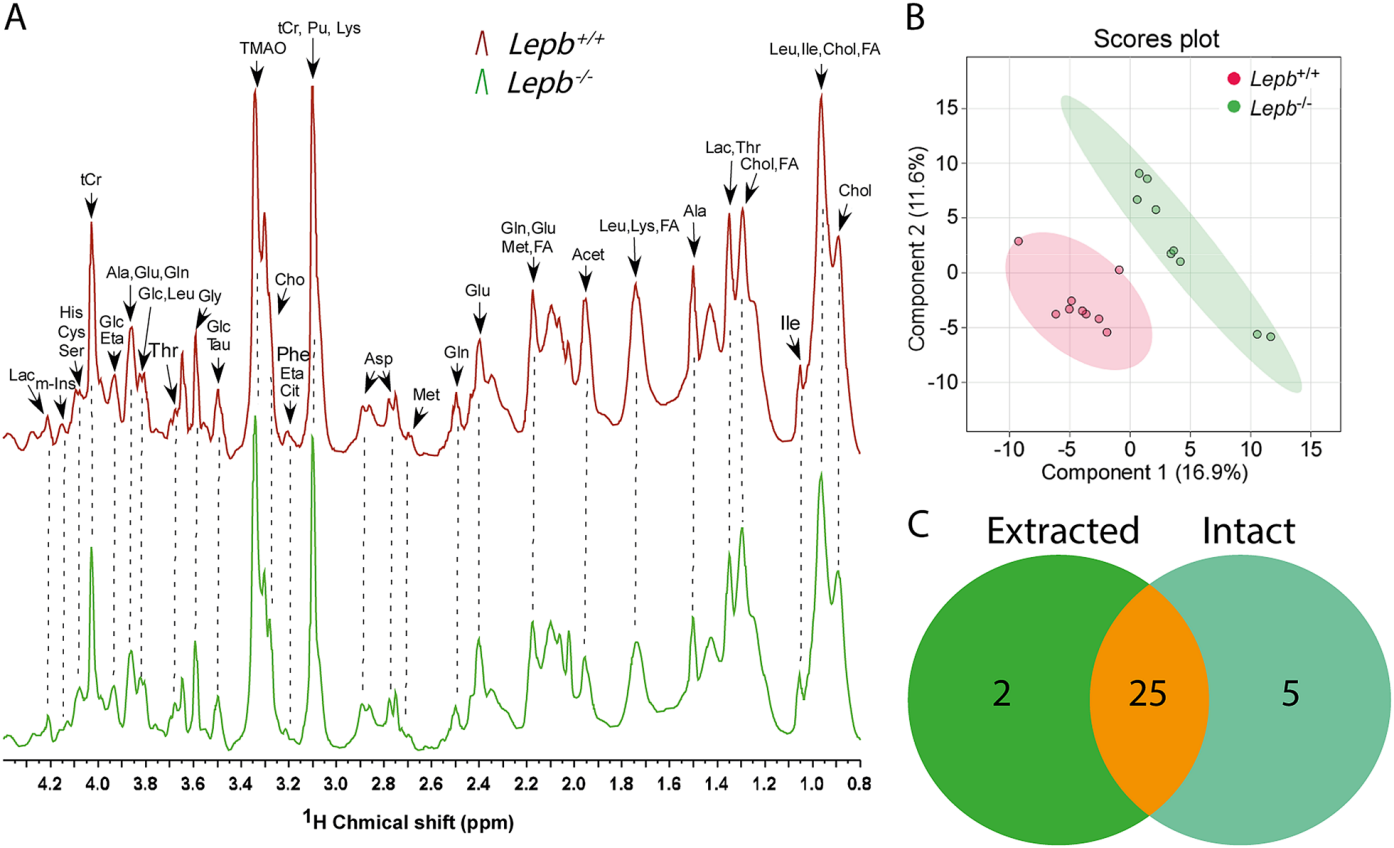
One-dimensional ^1^H HR-MAS NMR spectra and PLS-DA analysis of intact *lepb* mutant zebrafish larvae. **A** The representative spectra of intact larvae from wild type and *lepb* mutant groups measured by HR-MAS NMR spectrometry. Spectra from chemical shift 0.5–4.4 were assigned to specific metabolites. *Acet* acetate, *Ala* alanine, *Asp* aspartate, *Cho* choline, Chol cholesterol, *Cit* citrulline, *Cys* cysteine, *Eta* ethanolamine, *FA* fatty acid, *Glc* glucose, *Gln* glutamine, *Glu* glutamate, *Gly* glycine, *His* histidine, *Ile* isoleucine, *Lac* lactate, *Leu* leucine, *Lys* lysine, *Met* methionine, *m-Ins* myo-inositol, *Pu* putrescine, *Ser* serine, *Tau* taurine, *tCr* total creatine (creatine + phosphocreatine), *Thr* threonine, *TAMO* trimethylamine N-oxide, *HR-MAS NMR* high-resolution magic-angle-spinning nuclear magnetic resonance. **B** PLS-DA analysis of intact larvae from wild type and *lepb* mutant groups, n = 3, three times measurements, each replicate represents 120 pooled larvae. *PLS-DA* partial least square discriminant analysis. **C** A Venn diagram is shown of the common 25 metabolites that are significantly changed both in extracted zebrafish larvae measured by ^1^H solution NMR and intact larvae measured by ^1^H HR-MAS NMR

**Fig. 4:**
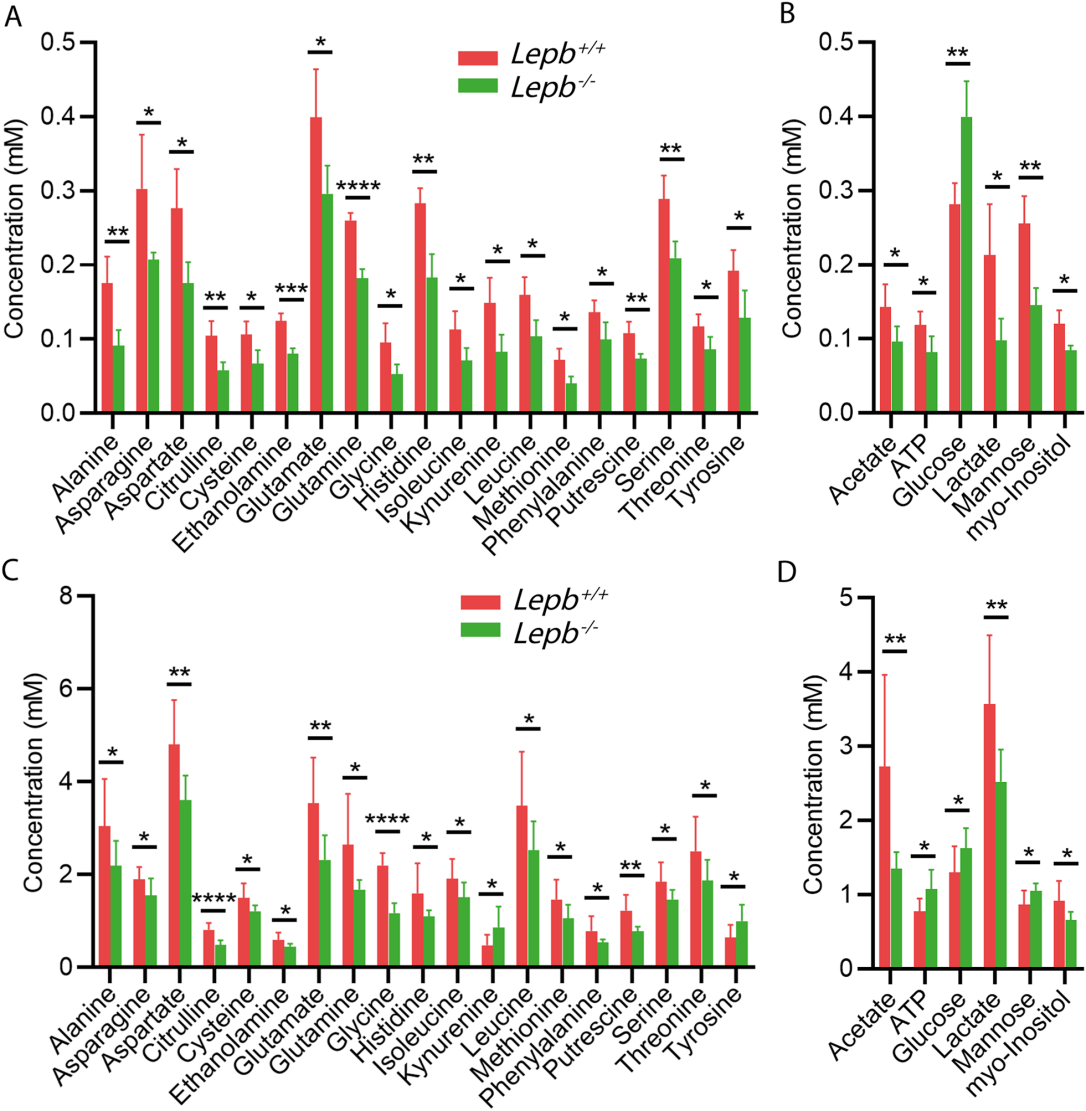
Quantification of the common 25 metabolites that are significantly changed in zebrafish larvae. **A** The concentration of amino acids and amines of wild type and *lepb* mutant in extracted larvae. **B** The concentration of ATP, carbohydrates and organic acids of wild type and *lepb* mutant in extracted larvae. **C** The concentration of amino acids and amines of wild type and *lepb* mutant in intact larvae. **D** The concentration of ATP, carbohydrates and organic acids of wild type and *lepb* mutant in intact larvae. *p < 0.05, **p < 0.01, ***p < 0.0005, ****p < 0.0001

Due to the possibility of degradation and selective loss of compounds because of the extraction method needed for solution NMR, we used HR-MAS NMR as a comparative method on intact zebrafish larvae. Figure 3A showed the comparison of metabolic profiles and the assignments of metabolites of representative spectra in *lepb* mutant and wild type siblings. It was shown that the intensities of many peaks were lower in the mutant group. A PLS-DA scores plot showed clear discrimination between the *lepb*^−/−^ and *lepb*^+/+^ groups (Fig. 3B). To compare the methods of solution NMR and HR-MAS NMR, we showed a Venn diagram of the significantly changed metabolites in the mutant and control siblings. The result revealed that there were 25 common metabolites significantly changed in both measurements (Fig. 3C). These 25 metabolites include the small amines alanine, asparagine, aspartate, citrulline, cysteine, ethanolamine, glutamate, glutamine, glycine, histidine, isoleucine, kynurenine, leucine, methionine, phenylalanine, putrescine, serine, threonine and tyrosine (Fig. 4A, C). In addition, the concentration of ATP, glucose, mannose, acetate, lactate and myo-inositol were changed significantly (Fig. 4B, D). For 21 of the 25 metabolites, both methods showed the same result: lower measurements of 20 metabolites and higher glucose level in the mutant group. However, kynurenine, tyrosine, ATP and mannose were detected at a decreased level in the mutant group with extracted larvae while at an increased level using intact larvae (Fig. 4).

### A core set of metabolites are markers for *leptin* deficiency in mice and zebrafish larvae

**Fig. 5:**
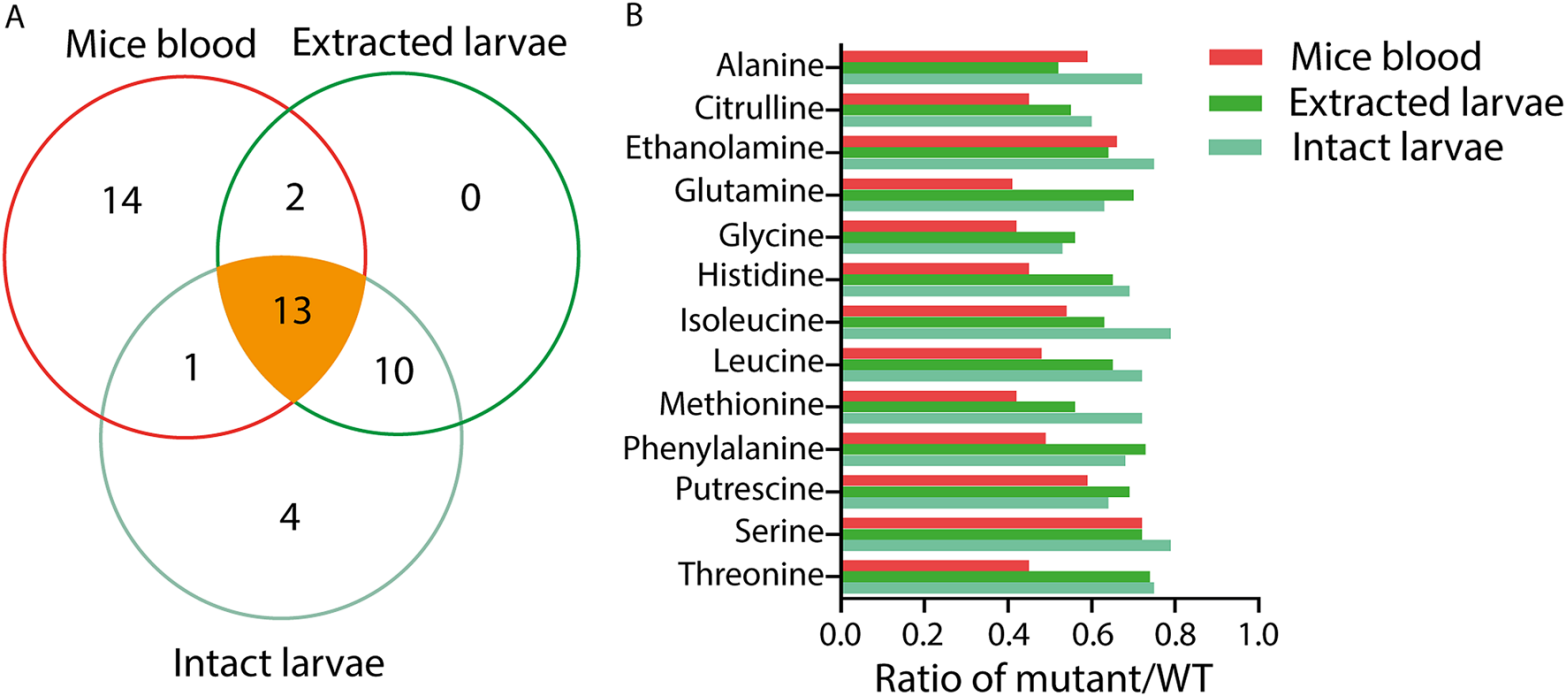
Common biomarkers for *leptin* deficiency in *ob/ob* mice, extracted and intact zebrafish larvae. **A** A Venn diagram shows that 13 common metabolites are significantly changed after leptin knockdown in mice blood, extracted and intact zebrafish larvae. **B** The ratio of leptin mutant versus wild type of the 13 common metabolites in the three metabolomic datasets

A common set of 13 metabolites were significantly changed in *ob/ob* mice blood, extracted *lepb* mutant and intact *lepb* mutant zebrafish larvae compared to their respective wild type controls (Fig. 5A). These 13 common metabolites were alanine, citrulline, ethanolamine, glutamine, glycine, histidine, isoleucine, leucine, methionine, phenylalanine, putrescine, serine and threonine (Fig. 5B). The concentrations of these 13 metabolites were reduced in a mutant compared to wild types for all the three metabolomic data sets (Fig. 5B). Of these metabolites, the following six are also reported as markers for tuberculosis infection in human, mice and zebrafish larvae based on mass spectrometry: citrulline, ethanolamine, leucine, methionine, phenylalanine, serine and threonine [26].

### Lipid profiles of *lepb*-deficient zebrafish larvae

**Fig. 6:**
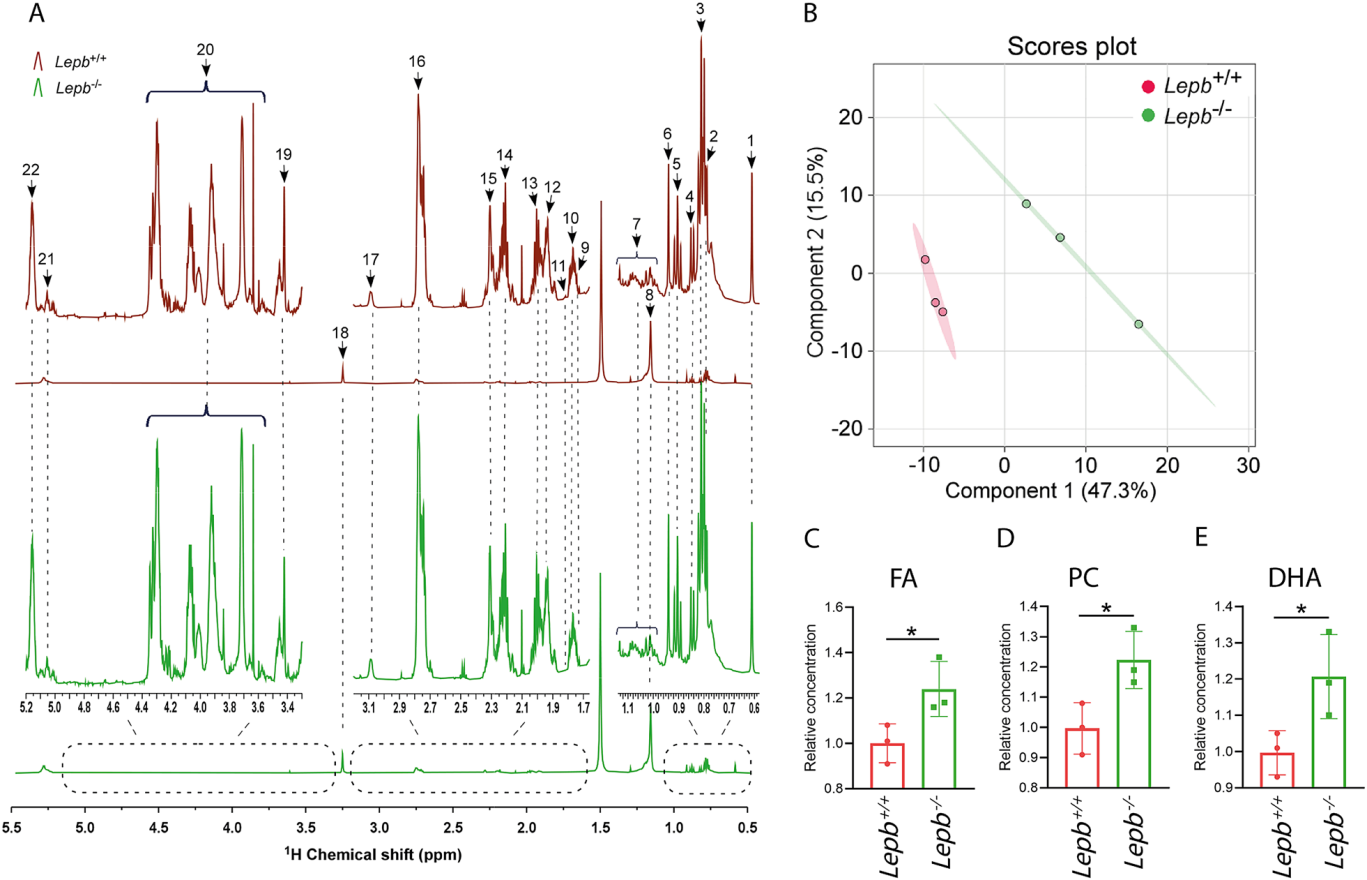
Lipid profiles of *lepb* mutant zebrafish larvae compared to wild type siblings. **A** The representative spectra of total lipid extracts from wild type and *lepb* mutant zebrafish larvae obtained by ^1^H NMR spectroscopy. The assignments of the peak numbers were shown in Additional file 1: Table S2. *NMR* nuclear magnetic resonance. **B** PLS-DA analysis of *lepb* mutant and wild type zebrafish larvae, n = 3, each replicate represents 105 pooled larvae. *PLS-DA* partial least square discriminant analysis. **C** The relative concentration of the signal 14 FA in **A**. *FA* fatty acids. **D** The relative concentration of the signal 18 PC in **A**. *PC* phosphatidylcholines. **E** The relative concentration of the signal 15 DHA in **A**. *DHA* docosahexaenoic acid. *p < 0.05

To investigate whether lipid metabolism is influenced by leptin deficiency at the early stage of zebrafish development, lipids were extracted from pooled 5 days post fertilization (dpf) zebrafish larvae in the *lepb* mutant and sibling control groups and then measured with ^1^H solution NMR (Fig. 6A). A PLS-DA scores plot of the tetramethylsilane (TMS) normalized spectra showed a clear separation of the lipid profiles of the two groups (Fig. 6B), which indicated lipid metabolism was altered in *lepb* mutant zebrafish larvae. Twenty-two lipid signals could be assigned from chemical shift 0.5–5.5 in the spectra of both groups (Fig. 6A, Additional file 1: Table S2). Based on the quantification of normalized peaks, we can conclude that saturated lipids were significantly increased in the *lepb* mutant zebrafish larvae (Fig. 6C, D). In addition, the polyunsaturated fatty acid (PUFA) docosahexaenoic acid (DHA) was found in a higher abundance in the mutant group (Fig. 6E).

### Deep sequencing of transcriptomes of *leptin* deficient mice and zebrafish larvae

**Fig. 7:**
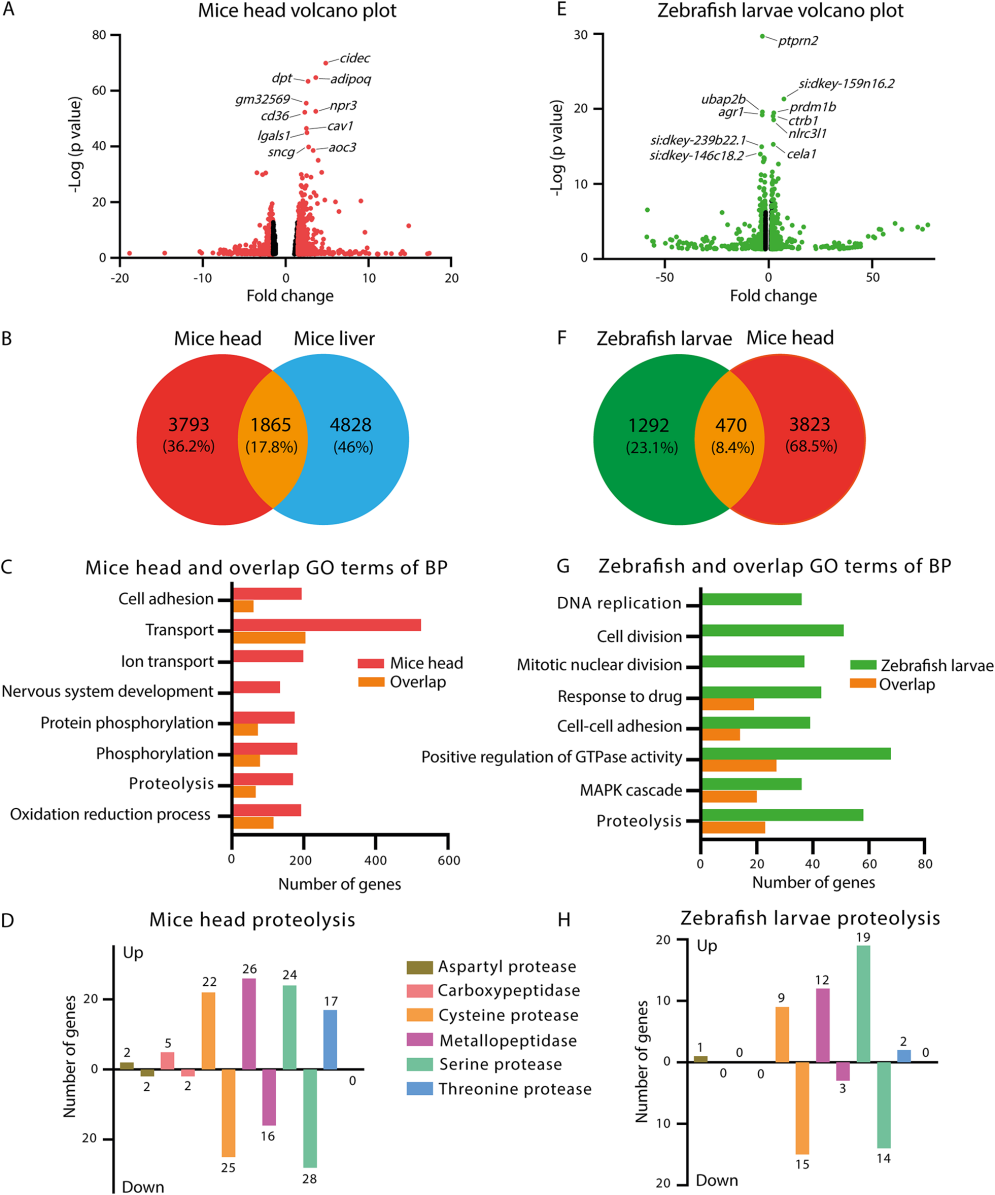
Transcriptome signature sets of mice and zebrafish larvae. **A** A Volcano plot showing a graphical representation of the significance (*p* < 0.05) in *ob/ob* mice head compared to C57BL/6 mice head. The transcripts with fold change over 1.5 are highlighted in red. Fifteen significant genes in mice head out of the fold change in X axis are excluded to make the graph look well. **B** A Venn diagram showing the comparison of the number of significantly changed genes between *ob/ob* mice head and mice liver published by Kokaji et al. **C** The top eight GO terms of biological process (BP) with lowest *p* adjusted values and highest numbers of genes representatives in mice head and the overlap of **B**. *GO* gene ontology. **D** Number of genes in classification of GO term proteolysis in the signature set of mice head. **E** A Volcano plot showing a graphical representation of the significance (*p* < 0.05) in *lepb* mutant zebrafish larvae compared to wild type siblings. The transcripts with fold change over 1.5 are highlighted in green. Twenty-two significant genes in zebrafish larvae out of the fold change in X axis are excluded to make the graph look well. **F** A Venn diagram showing the comparison of the number of significantly changed genes from human homologs of the signature gene sets of zebrafish larvae and *ob/ob* mice head. **G** The top eight GO terms of BP with lowest *p* adjusted values and highest numbers of genes representatives in zebrafish larvae and the overlap of **F**. **H** Number of genes in classification of GO term proteolysis in the signature set of zebrafish larvae

We investigated the effects of *leptin* deficiency at the transcriptome level in mice and zebrafish larvae by using deep RNA sequencing methods. Samples were taken from the same experimental groups as used for the metabolomic analysis described above. Mice heads were taken as a body part of interest because of the known classical signaling of leptin in the brain. A volcano plot showed that 5658 genes significantly regulated at a *p* value < 0.05 in *ob/ob* mice compared to wild type C57BL/6 mice (Fig. 7A). A recent paper published by Kokaji et al. reported the transcriptomes of mice liver from ten-week-old male *ob/ob* mutant and C57BL/6 wild type mice [13]. The comparison of the two mice liver groups showed 6693 genes significantly regulated at a *p* value < 0.05 (Additional file 1: Figure S4). The two gene sets encompassing 5658 and 6693 genes of the transcriptomes in mice head and mice liver, respectively, showed an overlap of 1865 genes (Fig. 7B). Gene ontology (GO) enrichment analysis using DAVID showed a large group of GO terms. In Fig. 7C, we showed a selected set of GO terms (biological process) with the lowest *p* adjusted values and the highest numbers of genes representatives. The GO term nervous system development was in line with the function of leptin in the brain. The GO enrichment of the overlap sets in Fig. 7B gave comparable results as with the mouse head GO terms, with the exception of ion transport and nervous system development (Fig. 7C). This could be explained by the relatively large number of neuronal cells in the head compared to liver.

For zebrafish larvae, there were 2718 genes significantly regulated at a *p* value < 0.05 in *lepb* mutant zebrafish larvae compared to wild type siblings (Fig. 7E). We validated the mRNA expression level of a few representative genes in the *lepb*^+/+^ and *lepb*^−/−^ zebrafish larvae with qPCR (Additional file 1: Figure S5). The human orthologs of this zebrafish larvae gene set and of the mice head transcriptome *ob/ob* signature set showed an overlap of 470 genes (Fig. 7F). The GO enrichment analysis of Fig. 7G showed the top eight GO terms (biological process) with lowest *p* adjusted values and highest numbers of genes representatives in the signature set of zebrafish larvae (Fig. 7G). The GO enrichment of the overlap set gave a similar result as in the zebrafish larvae terms with the exception of DNA replication, cell division and mitotic nuclear division. As shown in the Fig. 7C, G, one of the top GO terms in the signature set of mice heads, zebrafish larvae and the overlap was proteolysis. We also found the GO term proteolysis to be significantly enriched in the overlap of mice head and liver *ob/ob* signature set (Fig. 7C). The genes linked to this GO term were proteases which could be classified as aspartyl protease, carboxypeptidase, cysteine protease, metallopeptidase, serine protease, and threonine protease (Fig. 7D, H, Additional file 1: Table S3, S4). The pattern of the enriched gene numbers of those proteases in the signature sets of mice heads and zebrafish was similar in the up or down regulated groups (Fig. 7D, H).

**Fig. 8:**
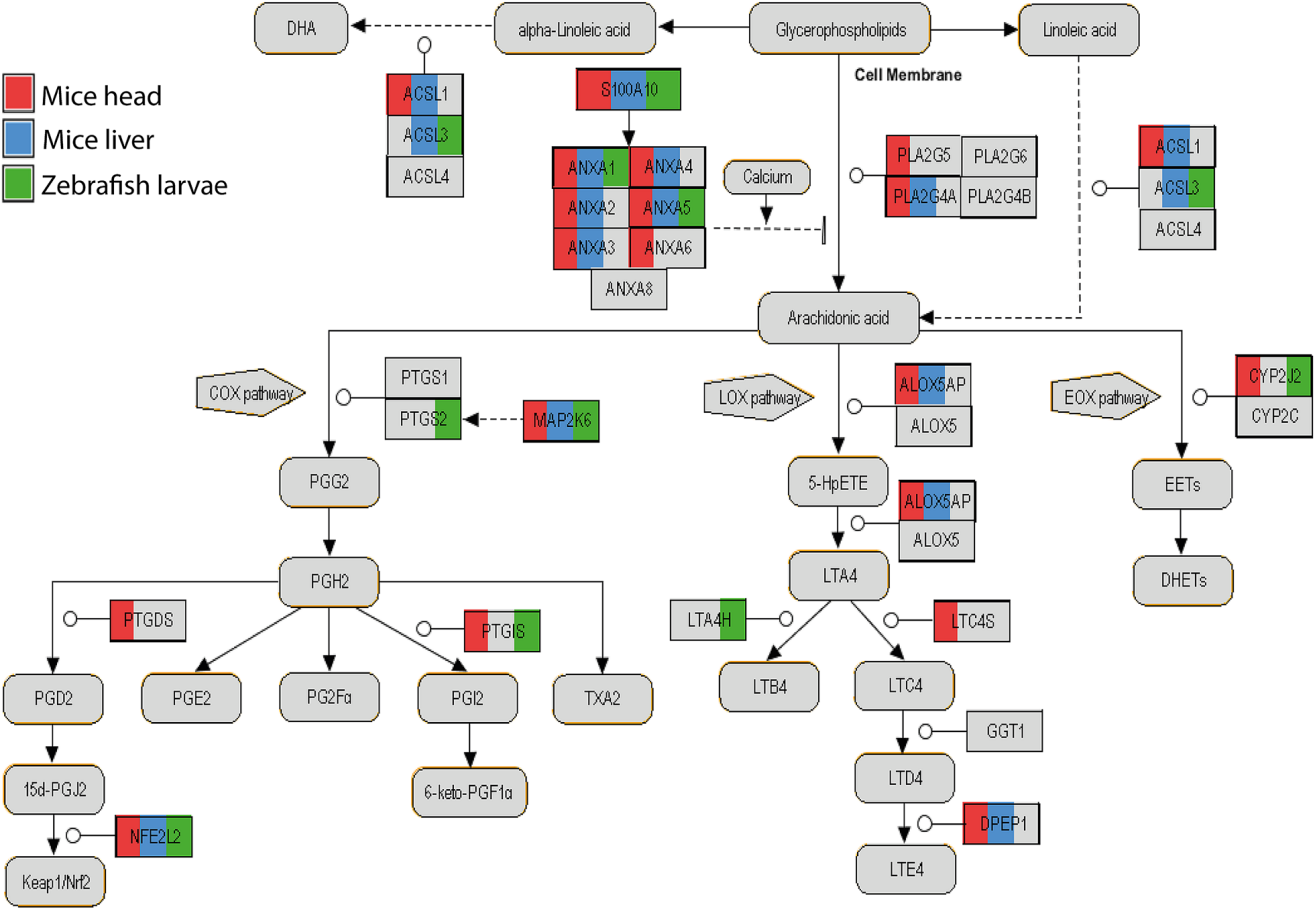
Genes involved in arachidonic acid pathway in human orthologs of the three transcriptome signature sets. Dashed lines means indirect regulation. Red color represents genes significantly changed in *ob/ob* mice head compared to control. Blue color represents genes significantly changed in *ob/ob* mice liver compared to wild type published by Kokaji et al. Green color represents genes significantly changed in *lepb* mutant zebrafish larvae compared to wild type siblings. *COX* cyclo-oxygenase, *LOX* lipoxygenase, *EOX* epoxygenase, *DHA* docosahexaenoic acid

As shown in Fig. 6, fatty acids such as DHA were significantly increased in the *lepb* mutant zebrafish larvae compared to wild type siblings. Lipid metabolism disturbance is possibly associated with inflammation [27]. Obese *leptin* deficient *ob/ob* mice show a low-grade chronic inflammation [28]. Interestingly, we found another common enriched GO term using DAVID (KEGG pathway) in the signature sets of mice head, mice liver and zebrafish larvae was arachidonic acid (ARA) metabolism (Additional file 1: Table S5–S7). Arachidonic acid is a pro-inflammatory precursor that can mediate inflammatory responses via transforming into a variety of downstream products such as prostaglandins and leukotrienes. It is also an early indicator of inflammation [27]. Therefore, the human orthologs of the signature sets of mice head, mice liver and zebrafish larvae were projected on the drawn ARA metabolic pathway based on the human wikipathways data using Pathvisio (Fig. 8). As shown in Fig. 8, five genes in the pathway namely *ANXA1, ANXA5, ACSL3, MAP2K6, NFE2L2* were altered in all three datasets. Some other genes were significantly changed in only one or two datasets. However, the majority of the gene expression levels of the three datasets visualized in this pathway were not high (Additional file 1: Table S8). This indicates there might be only mild inflammation in the *leptin* deficient mice and zebrafish larvae.

## Discussion

In this study, we have compared the metabolic changes resulting from *leptin* deficiency in blood of adult mice and extracted as well as intact zebrafish larvae. We studied metabolism using three different technologies: mass spectroscopy (MS), nuclear magnetic resonance (NMR) and high-resolution magic-angle-spinning NMR (HR-MAS NMR) spectrometry. In addition, we have compared the transcriptomic changes resulting from *leptin* deficiency in *ob/ob* mice heads and published data sets for *ob/ob* mice liver and *lepb* mutant zebrafish larvae using deep RNA sequencing (RNA-seq). These comparisons using very different omics technologies all show a remarkable similarity of the effects of leptin knockdown on the metabolomes and transcriptomes of adult mice and zebrafish larvae. These similarities are surprising because the analyzed samples of this comparative study are in many respects extremely different: (1) Mice and zebrafish are very diverse examples of the vertebrate subphylum, e.g., metabolic rate, body size, body temperature and examined life stages vary greatly; (2) Samples of blood or body tissue, in the case of the mice experiments, are compared with the entire organism in the case of zebrafish larvae; (3) The environmental conditions are different in mice and zebrafish larvae; (4) The genetic variation within the studied populations is highly diverse in zebrafish test samples, whereas a highly inbred population is used in the case of mice; and (5) For zebrafish larvae, there is no feeding of the organism involved and embryos are able to develop normally based on their reserves in the yolk until 5dpf. Nevertheless, also in a previous study, we showed remarkable similarities in small metabolite levels occurring in mice blood and zebrafish larvae after infection by mycobacteria [26]. The observed metabolic changes were mainly comprising a reduction of the levels of various amino acids that were also detected in human tuberculosis patients of several ethnical populations [26, 29, 30].

In the present study, we have also included HR-MAS NMR as a non-invasive method for analysis of metabolites in intact embryos. The results confirm the findings obtained with solution-state NMR analysis of extracted tissues. A few metabolites are changed in different directions measured by the two approaches, namely kynurenine, tyrosine, ATP and mannose. These are detected at a decreased level in the mutant group with extracted larvae while an increased level was detected with intact larvae using HR-MAS NMR (Fig. 4). This might be due to the fact that samples detected by solution-state NMR require extraction and pretreatment. Therefore, solubility with the used extraction solvents plays a key role in the detectable concentration. In addition, some metabolites might get degraded and oxidized during the extraction process. Conversely, these limitations are not present with HR-MAS NMR as it works with natural, unaltered, and intact samples at low temperature. Therefore, it likely better mirrors the underlying biochemical activity and state. In the case of kynurenine, this has been reported to have a significant higher level in blood of tuberculosis patients possibly due to an increased level of the enzyme indoleamine 2,3 dioxygenase 1 (IDO1) that converts tryptophan [29]. Tyrosine and mannose levels were previously also shown to be increased in mice and zebrafish samples using NMR analyses [26, 31]. Considering that zebrafish larvae and mouse and human blood samples are very similar in their metabolite profiles after mycobacterial infection [26], the increased level of kynurenine, tyrosine and mannose seen using HR-MAS NMR indicates an advantage of detecting metabolites directly in intact embryos using non-invasive HR-MAS NMR over extracted metabolites using solution NMR. However, a disadvantage of HR-MAS NMR compared to solution NMR is its lower resolution capacity for lipids.

As it is well known, rodents with leptin signaling deficiency show a typical phenotype of fat accumulation and obesity. Phospholipids and polyunsaturated fatty acids (PUFAs) including arachidonic acid and eicosapentaenoic acid are significantly increased in plasma and liver of *ob/ob* and *db/db* mice measured by MS [6]. Another study on obese Zucker and lean rats performed by ^1^H NMR reported increased concentrations of total fatty acids and triglycerides, while the ratio of PUFAs/monounsaturated fatty acids (MUFAs) was decreased in liver and blood of obese rats [11]. In our larval zebrafish *lepb* mutant, we also found that many lipid peaks are generally higher and for instance levels of DHA and phosphatidylcholines are significantly increased in *lepb* mutant larvae compared to the wild type siblings (Fig. 6). These observations demonstrate that *lepb* deficiency in zebrafish leads to lipid accumulation even at the organismal level at the larval stage. The parental adult *lepb* mutant zebrafish display distinctly more visceral fat compared to wild type sibling fish measured by magnetic resonance imaging (MRI) [32]. As zebrafish larvae before 5dpf only use yolk as their nutrition supply, which comes from the mother, zebrafish larvae offer a promising model to investigate maternal effects of the adult parents on the metabolic state of their offspring in the absence of a feeding regime. We reported previously that adult *lepb* mutant zebrafish display features of type 2 diabetes mellitus (T2DM) including higher glucose levels and develop early signs of diabetic nephropathy [32]. In this study, we also found that the concentration of glucose is significantly elevated in *lepb*^−/−^ zebrafish larvae compared to *lepb*^+/+^ group in both ^1^H NMR and HR-MAS NMR measurements. These observations in adult and larval zebrafish could lead to a better understanding of the effects of parents with gestational diabetes mellitus (GDM) on their offspring. GDM is one type of diabetes characterized by high blood pressure and high levels of glucose occurring only during pregnancy. Children from mothers suffering from GDM have a higher risk to develop obesity and T2DM, but also diabetic complications such as kidney disease. Unfortunately, it is impracticable to investigate maternal effects of GDM on offspring in humans and mammal animal models. Zebrafish larvae are therefore promising to explore the maternal effects of T2DM on their offspring as they develop outside the mother’s body [33, 34].

In this study, we demonstrate that 6 of the 13 amino acid metabolites of which the levels are reduced in both mutant *ob/ob* mice and *lepb*^*−/−*^ zebrafish larvae are also biomarkers for tuberculosis infection in human, mice and zebrafish larvae [26]. As it is well known, tuberculosis is also called a consumption disease with severe wasting syndrome symptoms at a later stage in TB patients. Therefore, the similarities between the deficiency of leptin and tuberculosis could be related to the occurrence of wasting syndrome in both *ob/ob* mice and *lepb* mutant zebrafish larvae. In this respect, metabolic changes due to *leptin* deficiency are also relevant for understanding T2DM that is accompanied by wasting syndrome. Of the 30 amino acids levels that we find reduced in the blood of *ob/ob* mice, several have been reported to be also changed in diabetic mice models in other studies. A decrease in glucogenic amino acids such as alanine, serine, glycine and glutamine indicates a high level of gluconeogenesis in *leptin* deficient animals. Plasma levels of glycine and serine were found to be significantly decreased in *ob/ob* mice and *db/db* mice compared to their wild type controls [6]. Leucine and isoleucine are two branched-chain amino acids (BCAAs) which are reported to stimulate protein synthesis in muscle [35, 36]. In contrast to our study, BCAAs levels were reported to be increased in *ob/ob* mice and *db/db* mice [6]. However, a study of human plasma samples demonstrated that the concentrations of the BCAAs, alanine and glutamine were significantly decreased in the plasma of T2DM patients compared to healthy volunteer groups [37]. The similarity of amino acid level changes resulting from *leptin* deficiency between mammals and zebrafish larvae provides the potential utility of common metabolites as biomarkers for both diabetic parents and their offspring by providing prognostic markers for the early identification of the risks of GDM.

The similarities in changes in metabolite levels resulting from *leptin* deficiency in different model organisms provide a way to further investigate the mechanism underlying these changes. In a first step towards further functional studies, we investigated the effect of *leptin* deficiency on the transcriptomic level. Studies have shown wasting syndrome to occur in obese animals as evidenced by muscle mass reduction due to the activation of proteolytic pathways such as the caspase-3 and the ubiquitin–proteasome proteolytic pathways [38, 39]. We also observed the gene ontology (GO) term proteolysis as one of the top GO terms in the transcriptome signature sets of *ob/ob* mice heads compared to wild type lean mice heads. This GO term was also enriched in the overlap set of this signature set with a signature set that we derived from a published liver transcriptome study of *ob/ob* mice compared to wild type mice (Fig. 7C). Genes involved in proteolysis can be classified as six types of proteases (Fig. 7D). Multiple proteolytic pathways are shown to be involved in wasting syndrome, including the following enzyme families: cysteine proteases such as calpains, cathepsins, caspases, ubiquitin peptidase families, metallopeptidases, serine proteases and threonine proteases such as proteasome subunit families [40]. Similar to the results obtained with the *ob/ob* mice body parts, we found that the expression levels of the genes encoding these proteases are significantly changed in *lepb* mutant zebrafish larvae compared to their wild type siblings (Fig. 7H). This is an indication that the *lepb* mutation leads to wasting syndrome even at an early stage of zebrafish larval development. It has been reported that amino acids are key regulators of protein turnover [41] and that the depletion of amino acids stimulates proteolysis in differentiated muscle cells [42]. The mechanisms underlying the observed reduced levels of amino acids in *ob/ob* mice and *lepb* mutant zebrafish larvae remains to be determined, but could be explained by protein degradation. The significant decrease of many amino acids in *ob/ob* mice and *lepb* mutant zebrafish might be a trigger for protein degradation to compensate for the loss of these amino acids.

In zebrafish larvae, both saturated fatty acids and polyunsaturated fatty acid DHA are increased in the *lepb* mutant group. DHA is an omega-3 fatty acid which is a precursor of eicosanoids such as resolvins and protectins with potential anti-inflammatory activity [43]. In contrast, omega-6 PUFA arachidonic acid (ARA) is a key precursor for eicosanoids such as prostaglandins, thromboxanes and leukotrienes which mediate inflammatory response [44]. Peak 11 of the spectra (Fig. 6A) could represent the PUFA arachidonic acid. However, the relatively low abundance and the overlap with the peak of eicosapentaenoic acid (EPA) made it hard to quantify the concentration in the two groups. In zebrafish larvae, genes such as *PTGS2*, *PTGIS*, involved in the generation of prostaglandins in the cyclo-oxygenase (COX) pathway are downregulated in *lepb*^−/−^ compared to the *lepb*^+/+^ group (Fig. 8, Additional file 1: Table S8). This might be the result of the anti-inflammatory effect of an increased level of DHA observed in *lepb* mutant zebrafish larvae. In *ob/ob* mice head and liver, genes like *PLA2G4A*, *ALOX5AP*, *DPEP1* involved in the release of ARA from cell membrane and lipoxygenase (LOX) pathway are significantly upregulated (Fig. 8, Additional file 1: Table S8). Therefore, more leukotrienes are expected to be produced, which leads to a potential inflammatory state. This is consistent with the generally accepted concept that obesity and type II diabetes are accompanied with chronic, low-grade inflammation [45]. This is in line with the previously shown correlation of leptin deficiency and diabetes with a higher susceptibility to tuberculosis [46]. Furthermore, it has been shown that zebrafish larvae and humans respond in a very similar way to infection with mycobacteria, for instance in the activation of the prostaglandin pathway [47, 48]. Therefore, the opportunities for future studies of the common mechanism underlying wasting syndrome in various disease such as T2DM and infectious disease in zebrafish larvae are extremely promising for leading to understand human diseases.

## Conclusion

*Leptin* deficiency in adult mice and larval zebrafish leads to highly similar metabolic alterations in amino acid levels. These metabolic changes show the same key features as observed during progression of tuberculosis in human patients, rodents and zebrafish larvae. This conclusion is supported by different technologies, namely MS, solution-state NMR and HR-MAS NMR. Moreover, by studying the transcriptome, we found highly similar changes in gene regulation related to proteolysis and arachidonic acid pathways in these two test systems. These results show a remarkable similarity of the effects of leptin knockdown on the metabolomes and transcriptomes of adult mice and zebrafish larvae that might be related to wasting syndrome. Apparently, the metabolic control by leptin is similar in adult and embryonic stages in mammals and fish, respectively.

## Materials and methods

### Biological materials

#### Mice

Male *ob/ob* mice and lean C57BL/6 wild type mice were obtained from Charles River Laboratories at 6 weeks of age (n = 8 per group) and maintained for 8 weeks under specific pathogen free conditions in the animal facility of the Leiden University Medical Center (LUMC). Male mice were chosen because metabolic variation due to the hormonal cycle is limited. Mice were kept on a standard-chow diet with ad libitum access to food and water. One *ob/ob* mouse had to be sacrificed at an early stage due to malocclusion. Body weight of all mice was measured weekly. Mice were sacrificed at week 14 and blood was collected and heads were snap-frozen in liquid nitrogen and stored at − 80 °C until RNA isolation. Mice heads were taken as a body part of interest because of the known classical signaling of leptin in the brain. Handling of mice was conducted in compliance with European Community Directive 86/609 for the care and use of laboratory animals and in accordance with the regulations set forward by the LUMC animal care committee.

### Mouse serum sample preparation

Mouse serum samples were collected from clotted blood tubes and mixed with pre-heated 80% ethanol at a 1:3 ratio (end concentration: 60% ethanol) in polypropylene screwcap tubes. Samples were heated for 10 min at 90 °C and subsequently chilled on ice for 10 min before centrifugation at 13.000 rpm for 10 min at 4 °C. Supernatants were harvested and stored at − 80 °C for LC–MS analysis.

### Zebrafish larvae

Zebrafish were handled in compliance with the local animal welfare regulations and maintained according to standard protocols (http://zfin.org). Mutant *lepb*^−/−^ and wild type sibling *lepb*^+/+^ zebrafish lines were generated, screened and raised as described previously [32]. A *lepb* mutant with a seven base pair deletion encompassing TAGAGGG in exon 2 was used in this study. Zebrafish larvae at 5 dpf from *lepb*^−/−^ and *lepb*^+/+^ groups were collected and stored at − 80℃ until further analysis. For solution-state NMR measurement, four replicate samples per genotype comprised of 105 pooled larvae were taken. From the same batch, three replicate samples per group of 15 pooled larvae were used for RNA isolation and transcriptome analysis. For HR-MAS NMR measurement, three replicates of 120 pooled larvae were used (each sample was measured three times).

### LC–MS/MS

Metabolite levels in mice serum were measured in individual replicates using a targeted LC–MS/MS platform as described before [26, 29]. Subject numbers were randomized and run in five batches which included a calibration line, QC samples and blanks. QC samples were analyzed every 10 samples. They were used to assess data quality and to correct for instrument responses.

The amine platform covers amino acids and biogenic amines employing an Accq-Tag derivatization strategy adapted from a previously published protocol [49]. Briefly, 5.0 μl of each sample was spiked with an internal standard solution. Then proteins were precipitated by the addition of MeOH. The supernatant was dried in a speedvac. The residue was reconstituted in borate buffer (pH 8.5) with AQC reagent. 1.0 μl of the reaction mixture was injected into the UPLC-MS/MS system. Chromatographic separation was achieved by an Agilent 1290 Infinity II LC System on an Accq-Tag Ultra column. The UPLC was coupled to electrospray ionization on a triple quadrupole mass spectrometer (AB SCIEX Qtrap 6500). Analytes were detected in the positive ion mode and monitored in Multiple Reaction Monitoring (MRM) using nominal mass resolution. Acquired data were evaluated using MultiQuant Software for Quantitative Analysis (AB SCIEX, Version 3.0.2). The data are expressed as relative response ratios (target area/ISTD area; unit free) using proper internal standards. For analysis of amino acids, their 13C15N-labeled analogs were used. For other metabolites, the closest-eluting internal standard was employed. In-house developed algorithms were applied using the pooled QC samples to compensate for shifts in the sensitivity of the mass spectrometer over the batches. After quality control correction, metabolite targets complied with the acceptance criteria of RSDqc  < 15%. Using this platform, we were able to identify 41 metabolites in blood samples from mice.

### MS data analysis

Data was analyzed using the software package MetaboAnalyst 4.0 [50]. MetaboAnalyst offers the possibility to provide automated data reports which we used for archiving data sets. Default settings were used with log transformation and auto scaling of the data for normalization. Naming of the metabolites is based on reference compounds using standard nomenclature of the human metabolome database (https://www.hmdb.ca/).

### ^1^H solution NMR measurement of extracted larvae

For ^1^H solution NMR spectroscopy, metabolites from pooled zebrafish larvae were extracted according to a previous study [26]. Zebrafish larvae were crushed and 1 ml mixture of methanol: water (1:1, v/v) and 1 ml chloroform were immediately added to the sample. The mixture was sonicated for 15 min and then centrifuged at 5000 rpm for 5 min. After centrifugation, two layers were formed: the upper layer is methanol and water containing metabolites, the lower layer is chloroform containing lipids. Those two layers were separately collected and evaporated via nitrogen gas flow. The metabolite pellets were resuspended in 600 μl of 100 mM deuterated phosphate buffer (KD2PO4, PH = 7.0) containing 0.02% trimethyl-silylpropanoic acid (TSP) as a reference and was subsequently centrifuged, and the supernatant was analyzed by solution NMR. The lipid pellets were resuspended in 600 μl deuterated chloroform containing 0.03% TMS which was used as a reference. Metabolites and lipids in zebrafish larvae were measured with a Bruker DMX 600 MHz NMR spectrometer at 4 °C equipped with a 5 mm inverse triple high-resolution probe with an actively shielded gradient coil. The ^1^H NMR spectra were accumulated with 65,000 data points, a 2-s relaxation delay, a sweep width of 12.4 kHz, and 256 scans which were required to obtain a satisfactory signal-to-noise ratio.

### ^1^H HR-MAS NMR measurement of intact larvae

Metabolic profiling by ^1^H HR-MAS NMR spectroscopy was performed as adapted from previous studies [51–53]. Zebrafish larvae from *lepb*^+/+^ and *lepb*^−/−^ groups were carefully transferred to a 4-mm zirconium oxide MAS NMR rotor (Bruker BioSpin AG, Switzerland). As a reference (^1^H chemical shift at 0 ppm), 10 µl of 100 mM deuterated phosphate buffer (KD_2_PO4, PH = 7.0) containing 0.1% (w/v) TSP was added to each sample. The rotor was then placed immediately inside the NMR spectrometer.

All HR-MAS NMR experiments were done on a Bruker DMX 600-MHz NMR spectrometer, which was equipped with a 4-mm HR-MAS dual inverse ^1^H/^13^C probe with a magic angle gradient and spinning rate of 6 kHz with a proton resonance frequency of 600 MHz. Measurements were carried out at a temperature of 277 K using a Bruker BVT3000 control unit. Acquisition and processing of data were done with Bruker TOPSPIN software 2.1 (Bruker Analytische Messtechnik, Germany).

A rotor synchronized Carr–Purcell–Meiboom–Gill (CPMG) pulse sequence with water suppression was used for one-dimensional ^1^H HR-MAS NMR spectra. Each one-dimensional spectrum was acquired applying a spectral width of 8000 Hz, domain data points of 16 k, a number of averages of 512 with 8 dummy scans, a constant receiver gain of 2048, an acquisition time of 2 s, and a relaxation delay of 2 s. The relaxation delay was set to a small value to remove nascent short transverse (*T*_*2*_) components due to the presence of lipids in intact embryo samples. All spectra were processed by an exponential window function corresponding to a line broadening of 1 Hz and zero-filled before Fourier transformation. NMR spectra were phased manually and automatically baseline corrected using TOPSPIN 2.1. The total analysis time (including sample preparation, optimization of NMR parameters, and data acquisition) of ^1^H HR-MAS NMR spectroscopy for each sample was approximately 20 min.

### NMR analysis

The one-dimensional ^1^H solution NMR and HR-MAS NMR spectra obtained from *lepb*^−/−^ and *lepb*^+/+^ group were corrected for baseline, phase shifts and reference using the MestReNova software version 11.0 (Mestrelab Research S.L., Santiago de Compostela, Spain). The region of 4.8–4.9 (solution NMR) was excluded from the analysis to remove the water peak. The spectra were then subdivided in the range between 0 and 10 ppm into buckets of 0.04 ppm. The resulting data matrix was saved as the format of script: NMR CSV matrix (transposed) (*.CSV, *.txt). This was then imported into MetaboAnalyst 4.0 for multivariate analysis using PLS-DA. Correlation coefficients with *p* < 0.05 were considered statistically significant. Quantification of metabolites was performed using Chenomx NMR Suite 8.6 (Edmonton, Alberta, Canada), which allowed for qualitative and quantitative analysis of an NMR spectrum by fitting spectral signatures from HMDB database to the respective spectrum. Assignment of peaks was based on the chemical shifts of compounds of interest in Chenomx software. The concentration of lipids was calculated by comparing the integral peak intensity of the lipids of interest with that of the reference TMS peak [54]. Statistical analysis (*t*-tests) of the NMR quantification results was performed with GraphPad Prism 8.0.1 (San Diego, CA, USA) and *p*-values < 0.05 were considered significant.

### RNA isolation

Frozen *ob/ob* and C57BL/6 mouse heads (n = 4) were thawed in 30 ml of TRIzol Reagent (Life Technologies) and manually crushed in a mortar while zebrafish larvae from *lepb*^+/+^ and *lepb*^−/−^ groups (n = 3) were resuspended and crushed in 0.5 ml of TRIzol Reagent. Subsequently, total RNA was extracted in accordance with the manufacturer’s instructions. Contaminating genomic DNA was removed using DNase I digestion for 15 min at 37 °C. RNA concentration was determined by NanoDrop 2000 (Thermo Scientific, the Netherlands). RNA integrity (RIN) was assessed by bioanalyzer (Agilent) and samples with RIN values > 6 were used for further library construction and sequencing.

### Deep sequencing

#### Mice

Deep sequencing of total RNA samples derived from *ob/ob* and lean C57BL/6 mice heads was performed at ZF-screens B.V. (Leiden, the Netherlands) as described in a previous study [55]. A total of 3 μg of RNA was used to generate RNA-seq libraries using the Illumina TruSeq RNA Sample Preparation Kit v2 (Illumina Inc., San Diego, USA). In the manufacturer’s instructions two modifications were made: In the adapter ligation step 1 μl instead of 2.5 μl adaptor was used; In the library size selection step, the library fragments were isolated using a double Ampure XP purification with a 0.7 × beads to library ratio. The resulting mRNA-seq libraries were sequenced using an Illumina HiSeq2000 instrument according to the manufacturer’s description with a read length of 50 nucleotides. Image analysis and base calling were done by the Illumina HCS version 1.15.1. At least 15 million reads were obtained that could be mapped to the mouse genome version GRCm38.

### Zebrafish larvae

Deep sequencing of the zebrafish larvae RNA from *lepb*^+/+^ and *lepb*^−/−^ groups was performed by GenomeScan B.V. (Leiden, the Netherlands). The NEBNext Ultra II Directional RNA Library Prep Kit for Illumina (NEB #E7760S/L) was used to process the samples. Briefly, mRNA was isolated from total RNA using oligo-dT magnetic beads. After fragmentation of the mRNA, a cDNA synthesis was performed. This was used for ligation of the sequencing adapters and PCR amplification of the resulting product. The quality and yield after sample preparation was measured with Fragment Analyzer. The size of the resulting products was consistent with the expected size distribution (a broad peak between 300 and 500 bp). Clustering and DNA sequencing using the NovaSeq6000 was performed according to manufacturer’s protocols. A concentration of 1.1 nM of DNA was used. For the zebrafish larval samples, data sets of paired end reads of 150 nucleotides were obtained with at least 20 million reads of reads that could be mapped to the zebrafish genome version GRCz11.

### Deep sequencing data mapping and analysis

Sequencing data of mice heads were aligned and mapped to the mouse genome GRCm38.p6 using Genetiles server [55]. Sequencing data of zebrafish larvae were aligned and mapped to the zebrafish genome GRCz11 using Salmon v1.2.1, and differential gene expression was analyzed using DESeq2 v1.21.1. Gene Ontology (GO) term enrichment and KEGG pathway analysis were performed in DAVID Bioinformatics Resources 6.8 (https://david.ncifcrf.gov/). The arachidonic acid pathway of Fig. 8 was drawn in Pathvisio software based on the wikipathways eicosanoid synthesis, eicosanoid metabolism via cytochrome P450 mono-oxygenases (CYP), prostaglandin synthesis, and omega3 and omega6 fatty acids synthesis [56]. Genes MAP2K6 and Nfe2l2 were added to the pathway based on literature [57, 58].

#### qPCR

Zebrafish larvae cDNA was generated from the same RNA samples of RNAseq by using iScript cDNA synthesis kit (Bio-Rad). qPCR experiment was performed by following a protocol of SsoAdvanced Universal SYBR^®^ Green Supermix kit (Bio-Rad). qPCR measurement was detected on a CFX96 machine (Bio-Rad). The Cq values of targeted genes were normalized to a zebrafish housekeeping gene *Tsp* as the expression level was not changed due to *lepb* mutation. The relative expression level were analyzed by using 2^−ΔΔCt^ method. We selected the representative genes based on the fold change, expression level, *p* adjusted value and the ease to make good primers. The forward and reverse primer sequences of tested genes in zebrafish larvae are showing below. *LO018181.1:* TGAAGCGACTGGGATGCTG/TGGATCTCTTCGTTCAAGGGTT. *Si:dkey-14d8.6*: ACTCCTATGATCAGCCCCTG/TTACAGCCAAACTCCCACACC. *Amy2al2*: AGCACAACCCAAACACGAAA/CTGAACTCCTCCATAGCCGT. *Tsp*: CCTGCCCATTTTCAGTC/TGTTGTTGCCTCTGTTGCTC.

## Supplementary Information

**Additional file 1: Figure S1. Body weight of ob/ob and wild type C57BL/6 mice from week 6 to week 14.** WT: Wild type. ****p < 0.0001**. Figure S2. Quantifications of the common biomarkers of the blood from ob/ob mice and wild type mice.** The original and normalized value of the 25 biomarkers showing in Fig. 1C are significantly (*p* < 0.05) decreased in *ob/ob* mice blood compared to wild type mice blood. WT: Wild type. **Figure S3. Quantifications of the common biomarkers from extracted lepb mutant zebrafish larvae and wild type siblings.** Quantifications of the common 19 biomarkers in Fig. 2C that are significantly changed in *lepb* mutant zebrafish larvae versus wild type. WT: Wild type. *p < 0.05, **p < 0.01, ***p < 0.0001. **Figure S4. A Volcano plot of published transcriptomes of mice liver.** A Volcano plot showing a graphical representation of the significance (*p* < 0.05) in *ob/ob* mice liver compared to C57BL/6 mice liver. The transcripts with fold change over 1.5 are highlighted in blue. Thirty-six significant genes in mice liver out of the fold change in X axis were excluded to make the graph look well. **Figure S5. Validation of gene mRNA expression level from RNAseq data in Zebrafish larvae using qPCR.** (A) Gene *lo018181.1*, ensembl code ENSDARG00000113971. (B) Gene *si:dkey-14d8.5*, ensembl code ENSDARG00000045835. (C) Gene *amy2al2*, ensembl code, ENSDARG00000009443. *p < 0.05, **p < 0.01. **Table S1. Ratio of metabolite quantities in blood of ob/ob mice compared to the control group.** The levels of 30 metabolites are significantly decreased in the *ob/ob* mice compared to the wild type C57BL/6 mice. **Table S2. Overview of assigned lipid signals in Fig. ****6**** from zebrafish larvae.** S: singlet, d: doublet, t: triplet, m: multiplet, quin: quintet; dd: double doublet, bs: broad singlet, bm: broad multiplet, Chol: cholesterol, EPA: eicosapentaenoic acid, AA: arachidonic acid, DHA: docosahexaenoic acid, FA: fatty acids, PC: phosphatidylcholine, PLs: phospholipids, PUFA: polyunsaturated fatty acid. **Table S3. Gene lists and classification of GO term proteolysis from transcriptomes of mice head. Table S4. Gene lists and classification of GO term proteolysis from transcriptomes of zebrafish larvae. Table S5. Gene lists of GO term arachidonic acid metabolism from transcriptomes of mice head**. **Table S6. Gene lists of GO term arachidonic acid metabolism from transcriptomes of published mice liver. Table S7. Gene lists of GO term arachidonic acid metabolism from transcriptomes of zebrafish larvae. Table S8. Gene signature sets of mice head, mice liver and zebrafish larvae in Fig. ****8****.**

## Data Availability

All data generated or analyzed during this study are included in this published article and its Additional file 1.

## Abbreviations

ARA: Arachidonic acid
BCAAs: Branched-chain amino acids
COX: Cyclo-oxygenase
DHA: Docosahexaenoic acid
dpf: Day post fertilization
EOX: Epoxygenase
EPA: Eicosapentaenoic acid
GDM: Gestational diabetes mellitus
GO: Gene ontology
HR-MAS NMR: High-resolution magic-angle-spinning NMR
lepa: Leptin a
lepb: Leptin b
lepr: Leptin receptor
LOX: Lipoxygenase
MRI: Magnetic resonance imaging
MS: Mass spectrometry
Mtb: *Mycobacterium tuberculosis*
MUFA: Monounsaturated fatty acid
NMR: Nuclear magnetic resonance
PLS-DA: Partial Least Squares Discriminant Analysis
PUFA: Polyunsaturated fatty acid
RNAseq: RNA sequencing
T2DM: Type 2 diabetes mellitus
TMS: Tetramethylsilane
TSP: Trimethyl-silylpropanoic acid
WT: Wild type

## References

[1] Zhang Y, Proenca R, Maffei M, Barone M, Leopold L, Friedman JM (1994). Positional cloning of the mouse obese gene and its human homologue. Nature.

[2] Perakakis N, Farr OM, Mantzoros CS (2021). Leptin in leanness and obesity: JACC state-of-the-art review. J Am Coll Cardiol.

[3] Paz-Filho G, Mastronardi C, Delibasi T, Wong M-L, Licinio J (2010). Congenital leptin deficiency: diagnosis and effects of leptin replacement therapy. Arq Bras Endocrinol Metab.

[4] Meehan CA, Cochran E, Kassai A, Brown RJ, Gorden P (2016). Metreleptin for injection to treat the complications of leptin deficiency in patients with congenital or acquired generalized lipodystrophy. Expert Rev Clin Pharmacol.

[5] Wang B, Chandrasekera PC, Pippin JJ (2014). Leptin- and leptin receptor-deficient rodent models: relevance for human type 2 diabetes. Curr Diabetes Rev.

[6] Giesbertz P, Padberg I, Rein D, Ecker J, Hofle AS, Spanier B (2015). Metabolite profiling in plasma and tissues of ob/ob and db/db mice identifies novel markers of obesity and type 2 diabetes. Diabetologia.

[7] Won EY, Yoon MK, Kim SW, Jung Y, Bae HW, Lee D (2013). Gender-specific metabolomic profiling of obesity in leptin-deficient ob/ob mice by 1H NMR spectroscopy. PLoS ONE.

[8] Gogiashvili M, Edlund K, Gianmoena K, Marchan R, Brik A, Andersson JT (2017). Metabolic profiling of ob/ob mouse fatty liver using HR-MAS (1)H-NMR combined with gene expression analysis reveals alterations in betaine metabolism and the transsulfuration pathway. Anal Bioanal Chem.

[9] Gipson GT, Tatsuoka KS, Ball RJ, Sokhansanj BA, Hansen MK, Ryan TE (2008). Multi-platform investigation of the metabolome in a leptin receptor defective murine model of type 2 diabetes. Mol Biosyst.

[10] Connor SC, Hansen MK, Corner A, Smith RF, Ryan TE (2010). Integration of metabolomics and transcriptomics data to aid biomarker discovery in type 2 diabetes. Mol Biosyst.

[11] Serkova NJ, Jackman M, Brown JL, Liu T, Hirose R, Roberts JP (2006). Metabolic profiling of livers and blood from obese Zucker rats. J Hepatol.

[12] Perry RJ, Wang Y, Cline GW, Rabin-Court A, Song JD, Dufour S (2018). Leptin mediates a glucose-fatty acid cycle to maintain glucose homeostasis in starvation. Cell.

[13] Kokaji T, Hatano A, Ito Y, Yugi K, Eto M, Morita K (2020). Transomics analysis reveals allosteric and gene regulation axes for altered hepatic glucose-responsive metabolism in obesity. Sci Signal.

[14] Gorissen M, Bernier NJ, Nabuurs SB, Flik G, Huising MO (2009). Two divergent leptin paralogues in zebrafish (*Danio rerio*) that originate early in teleostean evolution. J Endocrinol.

[15] Prokop JW, Duff RJ, Ball HC, Copeland DL, Londraville RL (2012). Leptin and leptin receptor: analysis of a structure to function relationship in interaction and evolution from humans to fish. Peptides.

[16] Araújo JR, Keating E, Martel F (2015). Impact of gestational diabetes mellitus in the maternal-to-fetal transport of nutrients. Curr Diab Rep.

[17] Guelfi KJ, Ong MJ, Li S, Wallman KE, Doherty DA, Fournier PA (2017). Maternal circulating adipokine profile and insulin resistance in women at high risk of developing gestational diabetes mellitus. Metabolism.

[18] Kampmann FB, Thuesen ACB, Hjort L, Bjerregaard AA, Chavarro JE, Frystyk J (2019). Increased leptin, decreased adiponectin and FGF21 concentrations in adolescent offspring of women with gestational diabetes. Eur J Endocrinol.

[19] Radaelli T, Varastehpour A, Catalano P, Hauguel-de MS (2003). Gestational diabetes induces placental genes for chronic stress and inflammatory pathways. Diabetes.

[20] Yamashita H, Shao J, Ishizuka T, Klepcyk PJ, Muhlenkamp P, Qiao L (2001). Leptin administration prevents spontaneous gestational diabetes in heterozygous Leprdb/+ mice: effects on placental leptin and fetal growth. Endocrinology.

[21] Audira G, Sarasamma S, Chen JR, Juniardi S, Sampurna BP, Liang ST (2018). Zebrafish mutants carrying leptin a (lepa) gene deficiency display obesity, anxiety, less aggression and fear, and circadian rhythm and color preference dysregulation. Int J Mol Sci.

[22] He J, Ding Y, Nowik N, Jager C, Eeza MNH, Alia A (2021). Leptin deficiency affects glucose homeostasis and results in adiposity in zebrafish. J Endocrinol.

[23] Michel M, Page-McCaw PS, Chen W, Cone RD (2016). Leptin signaling regulates glucose homeostasis, but not adipostasis, in the zebrafish. Proc Natl Acad Sci USA.

[24] Marín-Juez R, Jong-Raadsen S, Yang S, Spaink HP (2014). Hyperinsulinemia induces insulin resistance and immune suppression via Ptpn6/Shp1 in zebrafish. J Endocrinol.

[25] Menni C, Fauman E, Erte I, Perry JR, Kastenmuller G, Shin SY (2013). Biomarkers for type 2 diabetes and impaired fasting glucose using a nontargeted metabolomics approach. Diabetes.

[26] Ding Y, Raterink RJ, Marin-Juez R, Veneman WJ, Egbers K, van den Eeden S (2020). Tuberculosis causes highly conserved metabolic changes in human patients, mycobacteria-infected mice and zebrafish larvae. Sci Rep.

[27] Sztolsztener K, Chabowski A, Harasim-Symbor E, Bielawiec P, Konstantynowicz-Nowicka K (2020). Arachidonic acid as an early indicator of inflammation during non-alcoholic fatty liver disease development. Biomolecules.

[28] Sáinz N, Rodríguez A, Catalán V, Becerril S, Ramírez B, Gómez-Ambrosi J (2010). Leptin administration downregulates the increased expression levels of genes related to oxidative stress and inflammation in the skeletal muscle of *ob/ob* mice. Mediators Inflamm.

[29] Vrieling F, Alisjahbana B, Sahiratmadja E, van Crevel R, Harms AC, Hankemeier T (2019). Plasma metabolomics in tuberculosis patients with and without concurrent type 2 diabetes at diagnosis and during antibiotic treatment. Sci Rep.

[30] Weiner J, Maertzdorf J, Sutherland JS, Duffy FJ, Thompson E, Suliman S (2018). Metabolite changes in blood predict the onset of tuberculosis. Nat Commun.

[31] Shin J-H, Yang J-Y, Jeon B-Y, Yoon YJ, Cho S-N, Kang Y-H (2011). 1H NMR-based metabolomic profiling in mice infected with *Mycobacterium tuberculosis*. J Proteome Res.

[32] He J, Ding Y, Nowik N, Jager C, Eeza MNH, Alia A (2021). Leptin deficiency affects glucose homeostasis and results in adiposity in zebrafish. J Endocrinol.

[33] Pelegri F (2003). Maternal factors in zebrafish development. Dev Dyn.

[34] Lee MT, Bonneau AR, Giraldez AJ (2014). Zygotic genome activation during the maternal-to-zygotic transition. Annu Rev Cell Dev Biol.

[35] O'Connell TM (2013). The complex role of branched chain amino acids in diabetes and cancer. Metabolites.

[36] Anthony JC, Reiter AK, Anthony TG, Crozier SJ, Lang CH, MacLean DA (2002). Orally administered leucine enhances protein synthesis in skeletal muscle of diabetic rats in the absence of increases in 4E-BP1 or S6K1 phosphorylation. Diabetes.

[37] van Doorn M, Vogels J, Tas A, van Hoogdalem EJ, Burggraaf J, Cohen A (2007). Evaluation of metabolite profiles as biomarkers for the pharmacological effects of thiazolidinediones in type 2 diabetes mellitus patients and healthy volunteers. Br J Clin Pharmacol.

[38] Sishi B, Loos B, Ellis B, Smith W, du Toit EF, Engelbrecht AM (2011). Diet-induced obesity alters signalling pathways and induces atrophy and apoptosis in skeletal muscle in a prediabetic rat model. Exp Physiol.

[39] Wang X, Hu Z, Hu J, Du J, Mitch WE (2006). Insulin resistance accelerates muscle protein degradation: activation of the ubiquitin-proteasome pathway by defects in muscle cell signaling. Endocrinology.

[40] Pasiakos SM, Carbone JW (2014). Assessment of skeletal muscle proteolysis and the regulatory response to nutrition and exercise. IUBMB Life.

[41] Kadowaki M, Kanazawa T (2003). Amino acids as regulators of proteolysis. J Nutr.

[42] Bechet D, Tassa A, Combaret L, Taillandier D, Attaix D (2005). Regulation of skeletal muscle proteolysis by amino acids. J Ren Nutr.

[43] Yan Y, Jiang W, Spinetti T, Tardivel A, Castillo R, Bourquin C (2013). Omega-3 fatty acids prevent inflammation and metabolic disorder through inhibition of NLRP3 inflammasome activation. Immunity.

[44] Kuehl F, Egan R (1980). Prostaglandins, arachidonic acid, and inflammation. Science.

[45] Hotamisligil GS (2006). Inflammation and metabolic disorders. Nature.

[46] Dooley KE, Chaisson RE (2009). Tuberculosis and diabetes mellitus: convergence of two epidemics. Lancet Infect Dis.

[47] Tobin DM, Roca FJ, Ray JP, Ko DC, Ramakrishnan L (2013). An enzyme that inactivates the inflammatory mediator leukotriene b4 restricts mycobacterial infection. PLoS ONE.

[48] Tobin David M, Roca Francisco J, Oh Sungwhan F, McFarland R, Vickery Thad W, Ray John P (2012). Host genotype-specific therapies can optimize the inflammatory response to mycobacterial infections. Cell.

[49] Noga MJ, Dane A, Shi S, Attali A, van Aken H, Suidgeest E (2012). Metabolomics of cerebrospinal fluid reveals changes in the central nervous system metabolism in a rat model of multiple sclerosis. Metabolomics.

[50] Chong J, Xia J (2020). Using MetaboAnalyst 4.0 for metabolomics data analysis, interpretation, and integration with other omics data. Methods Mol Biol.

[51] Berry JP, Roy U, Jaja-Chimedza A, Sanchez K, Matysik J, Alia A (2016). High-resolution magic angle spinning nuclear magnetic resonance of intact zebrafish embryos detects metabolic changes following exposure to teratogenic polymethoxyalkenes from algae. Zebrafish.

[52] Roy U, Conklin L, Schiller J, Matysik J, Berry JP, Alia A (2017). Metabolic profiling of zebrafish (*Danio rerio*) embryos by NMR spectroscopy reveals multifaceted toxicity of beta-methylamino-l-alanine (BMAA). Sci Rep.

[53] Zuberi Z, Eeza MNH, Matysik J, Berry JP, Alia A (2019). Toxins.

[54] van Amerongen YF, Roy U, Spaink HP, de Groot HJ, Huster D, Schiller J (2014). Zebrafish brain lipid characterization and quantification by (1)H nuclear magnetic resonance spectroscopy and MALDI-TOF mass spectrometry. Zebrafish.

[55] Veneman WJ, de Sonneville J, van der Kolk KJ, Ordas A, Al-Ars Z, Meijer AH (2015). Analysis of RNAseq datasets from a comparative infectious disease zebrafish model using GeneTiles bioinformatics. Immunogenetics.

[56] Pico AR, Kelder T, van Iersel MP, Hanspers K, Conklin BR, Evelo C (2008). WikiPathways: pathway editing for the people. PLOS Biol.

[57] Itoh K, Mochizuki M, Ishii Y, Ishii T, Shibata T, Kawamoto Y (2004). Transcription factor Nrf2 regulates inflammation by mediating the effect of 15-deoxy-Δ^12,14^ prostaglandin J_2_. Mol Cell Biol.

[58] Oyeniran C, Tanfin Z (2011). MAPK14 cooperates with MAPK3/1 to regulate endothelin-1-mediated prostaglandin synthase 2 induction and survival in leiomyoma but not in normal myometrial cells1. Biol Reprod.

