# Supplementary material for: Metabolomic and transcriptomic profiling of adult mice and larval zebrafish leptin mutants reveal a common pattern of changes in metabolites and signaling pathways
Source: Cell Biosci. 2021 Jul 7;11:126. doi: 10.1186/s13578-021-00642-0 (PMC8265131; doi:10.1186/s13578-021-00642-0)
Supplement: Supplementary file 1 — Additional file 1: Figure S1. Body weight of ob/ob and wild type C57BL/6 mice from week 6 to week 14. WT: Wild type. ****p < 0.0001. Figure S2. Quantifications of the common biomarkers of the blood from ob/ob mice and wild type mice. The original and normalized value of the 25 biomarkers showing in Fig. 1C are significantly (p < 0.05) decreased in ob/ob mice blood compared to wild type mice blood. WT: Wild type. Figure S3. Quantifications of the common biomarkers from extracted lepb mutant zebrafish larvae and wild type siblings. Quantifications of the common 19 biomarkers in Fig. 2C that are significantly changed in lepb mutant zebrafish larvae versus wild type. WT: Wild type. *p < 0.05, **p < 0.01, ***p < 0.0001. Figure S4. A Volcano plot of published transcriptomes of mice liver. A Volcano plot showing a graphical representation of the significance (p < 0.05) in ob/ob mice liver compared to C57BL/6 mice liver. The transcripts with fold change over 1.5 are highlighted in blue. Thirty-six significant genes in mice liver out of the fold change in X axis were excluded to make the graph look well. Figure S5. Validation of gene mRNA expression level from RNAseq data in Zebrafish larvae using qPCR. (A) Gene lo018181.1, ensembl code ENSDARG00000113971. (B) Gene si:dkey-14d8.5, ensembl code ENSDARG00000045835. (C) Gene amy2al2, ensembl code, ENSDARG00000009443. *p < 0.05, **p < 0.01. Table S1. Ratio of metabolite quantities in blood of ob/ob mice compared to the control group. The levels of 30 metabolites are significantly decreased in the ob/ob mice compared to the wild type C57BL/6 mice. Table S2. Overview of assigned lipid signals in Fig. 6 from zebrafish larvae. S: singlet, d: doublet, t: triplet, m: multiplet, quin: quintet; dd: double doublet, bs: broad singlet, bm: broad multiplet, Chol: cholesterol, EPA: eicosapentaenoic acid, AA: arachidonic acid, DHA: docosahexaenoic acid, FA: fatty acids, PC: phosphatidylcholine, PLs: phospholipids, PUFA: polyunsaturated fatty [file 13578_2021_642_MOESM1_ESM.docx]

**Additional file 1**

**Figure S1**


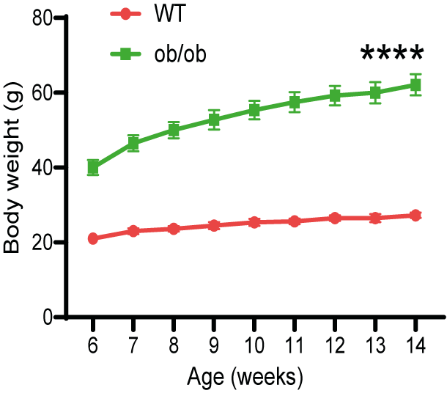


**Figure S1. Body weight of *ob/ob* and wild type C57BL/6 mice from week 6 to week 14.** WT: Wild type. *****p* < 0.0001**.**

**Figure S2**


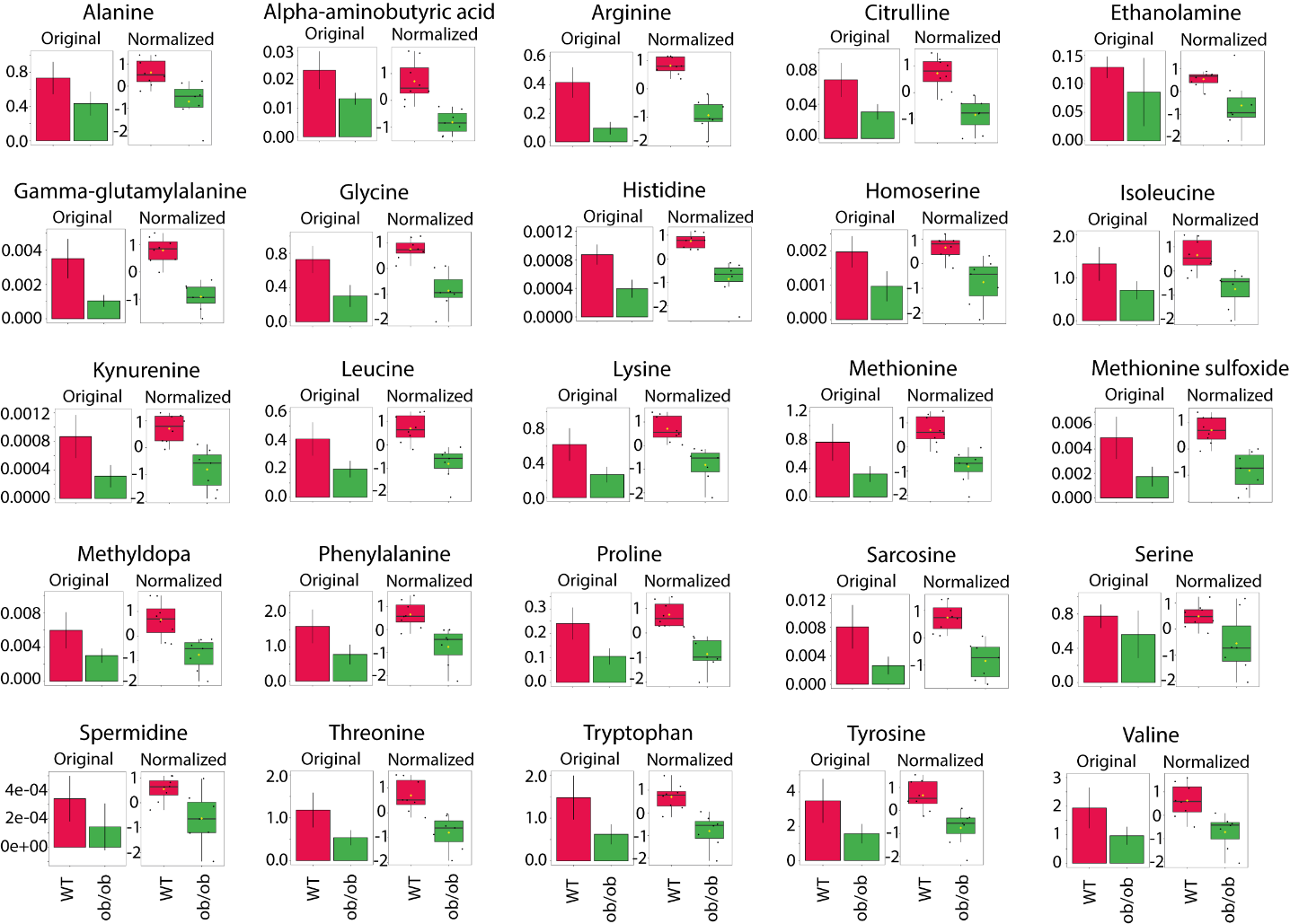


**Figure S2: Quantifications of the common biomarkers of the blood from *ob/ob* mice and wild type mice.** The original and normalized value of the 25 biomarkers showing in Figure 1C were significantly (p<0.05) decreased in *ob/ob* mice blood compared to wild type mice blood. Sample normalization was performed automatically by chosen log transformation and auto scaling in MetaboAnalyst 4.0. WT: Wild type.

**Figure S3**

**
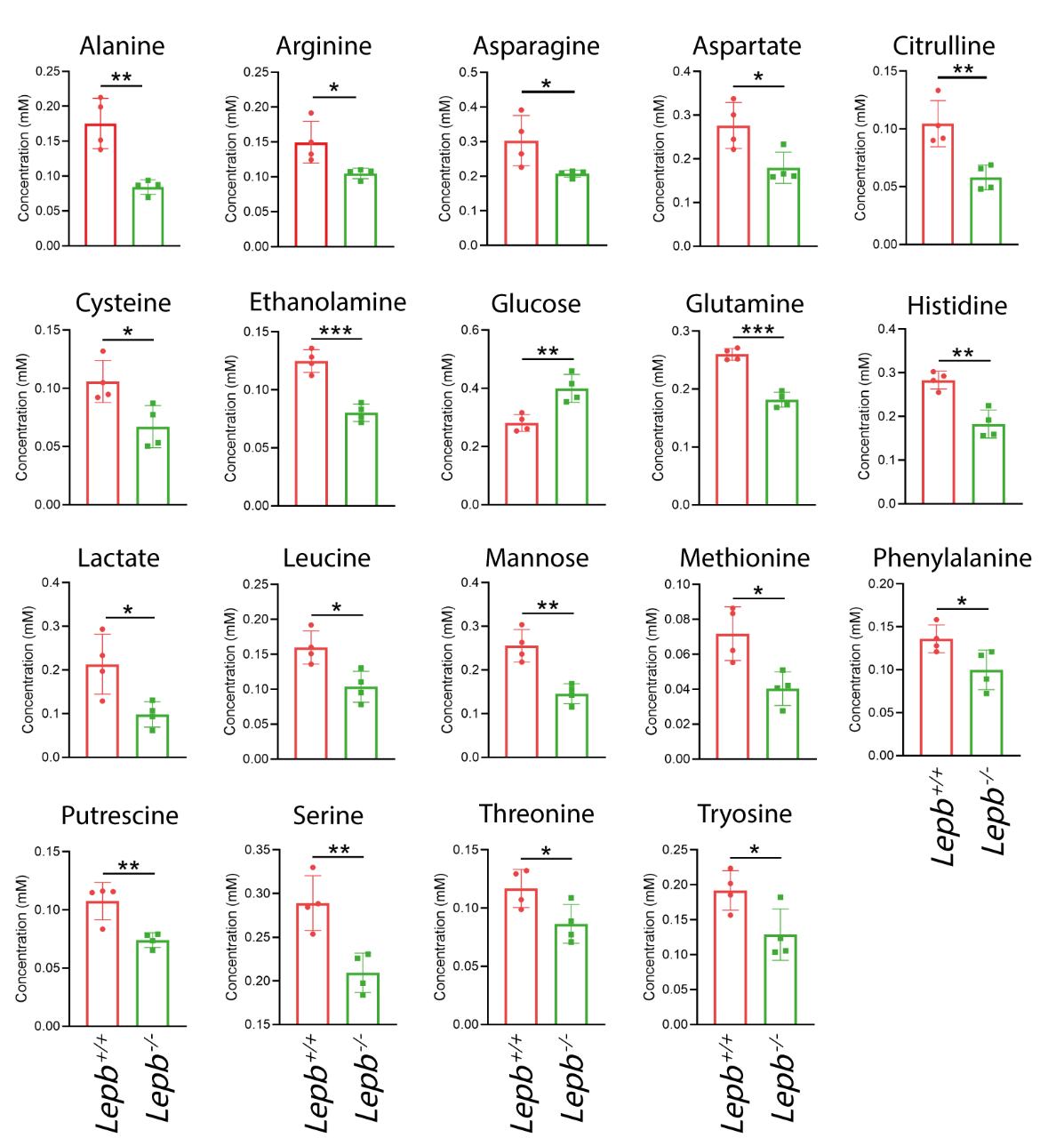
**

**Figure S3. Quantifications of the common biomarkers from extracted *lepb* mutant zebrafish larvae and wild type siblings.** Quantifications of the common 19 biomarkers in Figure 2C that are significantly changed in *lepb* mutant zebrafish larvae versus wild type. WT: Wild type. **p*<0.05, ***p*<0.01, ****p*<0.0001.

**Figure S4**


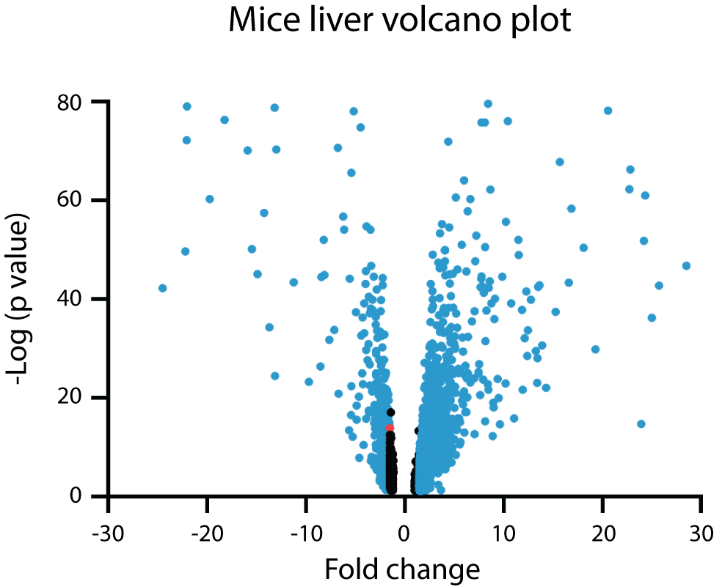


**Figure S4. A Volcano plot of published transcriptomes of mice liver.** A Volcano plot showing a graphical representation of the significance (*p*<0.05) in *ob/ob* mice liver compared to C57BL/6 mice liver. The transcripts with fold change over 1.5 are highlighted in blue. Thirty-six significant genes in mice liver out of the fold change in X axis were excluded to make the graph look well.

**Figure S5**

**
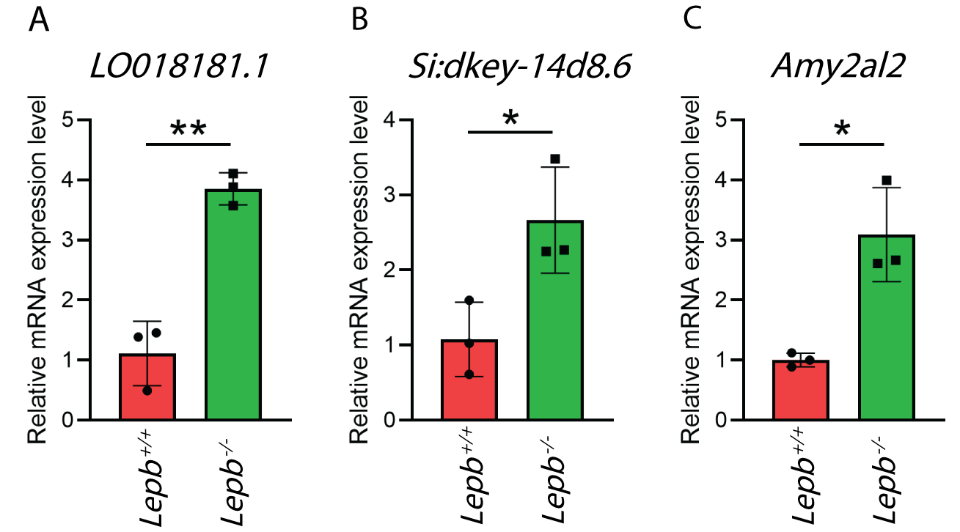
**

**Figure S5. Validation of gene mRNA expression level from RNAseq data in Zebrafish larvae using qPCR. A.** Gene *LO018181.1*, ensembl code ENSDARG00000113971**. B**. Gene *Si:dkey-14d8.5*, ensembl code ENSDARG00000045835. **C.** Gene *Amy2al2*, ensembl code, ENSDARG00000009443. **p*<0.05, ***p*<0.01.

**Table S1**

| **Metabolite** | **HMDB code** | **FC** | **P value** |
| --- | --- | --- | --- |
| Arginine | HMDB00517 | 0.23 | 3.00E-06 |
| Hydroxyproline | HMDB00725 | 0.29 | 6.00E-06 |
| Gamma-glutamylalanine | HMDB06248 | 0.30 | 1.00E-05 |
| Glutamine | HMDB00641 | 0.41 | 5.00E-05 |
| Histidine | HMDB00177 | 0.45 | 1.00E-04 |
| Glycine | HMDB00123 | 0.42 | 1.00E-04 |
| Proline | HMDB00162 | 0.44 | 1.00E-04 |
| Sarcosine | HMDB00271 | 0.33 | 2.00E-04 |
| Citrulline | HMDB00904 | 0.45 | 3.00E-04 |
| Methionine sulfoxide | HMDB02005 | 0.36 | 3.00E-04 |
| 2-Aminobutyrate | HMDB00510 | 0.32 | 4.00E-04 |
| Lysine | HMDB00182 | 0.43 | 5.00E-04 |
| Alpha-aminobutyric acid | HMDB00452 | 0.57 | 5.00E-04 |
| Methionine | HMDB00696 | 0.42 | 5.00E-04 |
| Kynurenine | HMDB00183 | 0.36 | 5.00E-04 |
| Tryptophan | HMDB00929 | 0.42 | 6.00E-04 |
| Hydroxylysine | HMDB00450 | 0.46 | 7.00E-04 |
| Leucine | HMDB00687 | 0.48 | 7.00E-04 |
| Threonine | HMDB00167 | 0.45 | 8.00E-04 |
| Tyrosine | HMDB00158 | 0.45 | 2.00E-03 |
| Methyldopa | HMDB11754 | 0.50 | 2.00E-03 |
| Phenylalanine | HMDB00159 | 0.49 | 2.00E-03 |
| Isoleucine | HMDB00172 | 0.54 | 2.00E-03 |
| Homoserine | HMDB00719 | 0.49 | 2.00E-03 |
| Valine | HMDB00883 | 0.50 | 4.00E-03 |
| Alanine | HMDB00161 | 0.59 | 5.00E-03 |
| Spermidine | HMDB01257 | 0.42 | 2.00E-02 |
| Ethanolamine | HMDB00149 | 0.66 | 2.00E-02 |
| Putrescine | HMDB01414 | 0.59 | 2.00E-02 |
| Serine | HMDB00187 | 0.72 | 4.00E-02 |

**Table S1. Ratio of metabolite quantities in blood of *ob/ob* mice compared to the control group.** The levels of 30 metabolites are significantly decreased in the *ob/ob* mice compared to the wild type C57BL/6 mice.

**Table S2**

| **Peak no.** | **Assignments** | **Zebrafish larvae** | |
| --- | --- | --- | --- |
|  |  | **Multiplicity** | **Chemical shifts (ppm) 600MHz** |
| 1 | Chol (C18) | s | 0.62 .. 0.60 |
| 2 | Chol (C26,27) | dd | 0.81 .. 0.78 |
| 3 | w-6 FA -CH3 (terminal) | d | 0.83 .. 0.81 |
| 4 | Chol (C21) | d | 0.86 .. 0.83 |
| 5 | w-3 FA -CH3 (terminal) | t | 0.92 .. 0.89 |
| 6 | Chol (C19) | s | 0.95 .. 0.93 |
| 7 | Chol | m | 1.12 .. 0.98 |
| 8 | Chol+FA (CH2) | s | 1.28 .. 1.15 |
| 9 | Chol+FA (CH2) | m | 1.76 .. 1.73 |
| 10 | FA (CH2) | bs | 1.80 .. 1.76 |
| 11 | Chol+FA (CH2) | d | 1.89 .. 1.88 |
| 12 | FA (CH2)–Chol | m | 1.96 .. 1.91 |
| 13 | FA (CH2) | quin | 2.05 .. 1.96 |
| 14 | FA (CH2)–Chol | dt | 2.25 .. 2.19 |
| 15 | FA (CH2)–DHA | m | 2.33 .. 2.28 |
| 16 | FA (CH2)–PUFA | dd | 2.81 .. 2.72 |
| 17 | PLs | bs | 3.13 .. 3.05 |
| 18 | PC | s | 3.29 .. 3.26 |
| 19 | Chol (C3) | m | 3.49 .. 3.41 |
| 20 | Phosphotidylglycerol | bm | 4.38 .. 3.55 |
| 21 | Sphingolipids | m | 5.11 .. 4.99 |
|  | Dolichols |  |  |
|  | Plasmalogens |  |  |
| 22 | FA (-CH = CH-) and Chol | m | 5.19 .. 5.12 |

**Table S2. Overview of assigned lipid signals in Figure 6 from zebrafish larvae.** S: singlet, d: doublet, t: triplet, m: multiplet, quin: quintet; dd: double doublet, bs: broad singlet, bm: broad multiplet, Chol: cholesterol, EPA: eicosapentaenoic acid, AA: arachidonic acid, DHA: docosahexaenoic acid, FA: fatty acids, PC: phosphatidylcholine, PLs: phospholipids, PUFA: polyunsaturated fatty acid.

**Table S3**

| **Mice ID** | **Mice gene name** | **Human homolog** | **Meas/Ctrl or -Ctrl/Meas (scaled)** | **p-value** | **Classification** |
| --- | --- | --- | --- | --- | --- |
| ENSMUSG00000070645 | Ren1 | ENSG00000143839 | -2.89 | 8.16E-04 | Aspartic peptidase |
| ENSMUSG00000032086 | Bace1 | ENSG00000186318 | -1.17 | 1.60E-03 | Aspartic peptidase |
| ENSMUSG00000007891 | Ctsd | ENSG00000117984 | 1.11 | 3.43E-02 | Aspartic peptidase |
| ENSMUSG00000058499 | Pip |  | 1.48 | 4.56E-02 | Aspartic peptidase |
| ENSMUSG00000039070 | Cpa4 | ENSG00000128510 | -2.67 | 3.05E-02 | Carboxypeptidase |
| ENSMUSG00000020841 | Cpd | ENSG00000108582 | -1.28 | 4.34E-05 | Carboxypeptidase |
| ENSMUSG00000039007 | Cpq | ENSG00000104324 | 1.22 | 4.13E-03 | Carboxypeptidase |
| ENSMUSG00000020473 | Aebp1 | ENSG00000106624 | 1.24 | 8.91E-03 | Carboxypeptidase |
| ENSMUSG00000027408 | Cpxm1 | ENSG00000088882 | 1.40 | 2.04E-05 | Carboxypeptidase |
| ENSMUSG00000001865 | Cpa3 | ENSG00000163751 | 1.90 | 9.45E-08 | Carboxypeptidase |
| ENSMUSG00000036596 | Cpz | ENSG00000109625 | 2.20 | 1.20E-07 | Carboxypeptidase |
| ENSMUSG00000034342 | Cbl | ENSG00000110395 | -1.64 | 8.07E-06 | Cysteine peptidase |
| ENSMUSG00000037326 | Capn15 | ENSG00000103326 | -1.35 | 9.53E-04 | Cysteine peptidase |
| ENSMUSG00000022637 | Cblb | ENSG00000114423 | -1.18 | 1.47E-02 | Cysteine peptidase |
| ENSMUSG00000026509 | Capn2 | ENSG00000162909 | 1.12 | 2.24E-02 | Cysteine peptidase |
| ENSMUSG00000001794 | Capns1 | ENSG00000126247 | 1.12 | 3.57E-02 | Cysteine peptidase |
| ENSMUSG00000021939 | Ctsb | ENSG00000164733 | 1.15 | 4.73E-04 | Cysteine peptidase |
| ENSMUSG00000083282 | Ctsf | ENSG00000174080 | 1.15 | 2.17E-02 | Cysteine peptidase |
| ENSMUSG00000028015 | Ctso | ENSG00000256043 | 1.25 | 1.63E-04 | Cysteine peptidase |
| ENSMUSG00000032359 | Ctsh | ENSG00000103811 | 1.25 | 4.55E-03 | Cysteine peptidase |
| ENSMUSG00000038642 | Ctss | ENSG00000163131 | 1.27 | 4.31E-04 | Cysteine peptidase |
| ENSMUSG00000054083 | Capn12 | ENSG00000182472 | 1.30 | 3.85E-03 | Cysteine peptidase |
| ENSMUSG00000016256 | Ctsz | ENSG00000101160 | 1.31 | 6.93E-05 | Cysteine peptidase |
| ENSMUSG00000079418 | Atg4a | ENSG00000101844 | 1.33 | 1.47E-02 | Cysteine peptidase |
| ENSMUSG00000025888 | Casp1 | ENSG00000137752 | 1.37 | 4.14E-02 | Cysteine peptidase |
| ENSMUSG00000021477 | Ctsl | ENSG00000136943 | 1.45 | 1.07E-11 | Cysteine peptidase |
| ENSMUSG00000033538 | Casp4 | ENSG00000137757 | 1.96 | 3.68E-03 | Cysteine peptidase |
| ENSMUSG00000028111 | Ctsk | ENSG00000143387 | 2.36 | 9.17E-08 | Cysteine peptidase |
| ENSMUSG00000035606 | Ky | ENSG00000174611 | -1.89 | 1.10E-08 | Cysteine peptidase |
| ENSMUSG00000090115 | Usp49 | ENSG00000164663 | -1.33 | 6.72E-04 | Cysteine peptidase |
| ENSMUSG00000031010 | Usp9x | ENSG00000124486 | -1.31 | 7.73E-05 | Cysteine peptidase |
| ENSMUSG00000051306 | Usp42 | ENSG00000106346 | -1.29 | 3.12E-03 | Cysteine peptidase |
| ENSMUSG00000056900 | Usp13 | ENSG00000058056 | -1.28 | 3.14E-04 | Cysteine peptidase |
| ENSMUSG00000042444 | Fam63b | ENSG00000128923 | -1.26 | 1.10E-05 | Cysteine peptidase |
| ENSMUSG00000051527 | Usp29 | ENSG00000131864 | -1.25 | 7.69E-03 | Cysteine peptidase |
| ENSMUSG00000033909 | Usp36 | ENSG00000055483 | -1.23 | 2.77E-03 | Cysteine peptidase |
| ENSMUSG00000028514 | Usp24 | ENSG00000162402 | -1.20 | 8.01E-04 | Cysteine peptidase |
| ENSMUSG00000056342 | Usp34 | ENSG00000115464 | -1.19 | 1.38E-03 | Cysteine peptidase |
| ENSMUSG00000036712 | Cyld | ENSG00000083799 | -1.17 | 1.01E-02 | Cysteine peptidase |
| ENSMUSG00000062627 | Mysm1 | ENSG00000162601 | -1.17 | 3.83E-02 | Cysteine peptidase |
| ENSMUSG00000040455 | Usp45 | ENSG00000123552 | -1.16 | 3.76E-02 | Cysteine peptidase |
| ENSMUSG00000045210 | Vcpip1 | ENSG00000175073 | -1.16 | 2.16E-02 | Cysteine peptidase |
| ENSMUSG00000043411 | Usp48 | ENSG00000090686 | -1.16 | 4.90E-03 | Cysteine peptidase |
| ENSMUSG00000054814 | Usp46 | ENSG00000109189 | -1.15 | 1.43E-02 | Cysteine peptidase |
| ENSMUSG00000038495 | Otud7b | ENSG00000264522 | -1.15 | 2.14E-02 | Cysteine peptidase |
| ENSMUSG00000052917 | Senp7 | ENSG00000138468 | -1.13 | 4.32E-02 | Cysteine peptidase |
| ENSMUSG00000020124 | Usp15 | ENSG00000135655 | -1.12 | 4.54E-02 | Cysteine peptidase |
| ENSMUSG00000027363 | Usp8 | ENSG00000138592 | -1.10 | 3.45E-02 | Cysteine peptidase |
| ENSMUSG00000027364 | Usp50 | ENSG00000170236 | -1.10 | 4.55E-02 | Cysteine peptidase |
| ENSMUSG00000019124 | Scrn1 | ENSG00000136193 | -1.10 | 3.06E-02 | Cysteine peptidase |
| ENSMUSG00000028964 | Park7 | ENSG00000116288 | 1.12 | 3.67E-02 | Cysteine peptidase |
| ENSMUSG00000028560 | Usp1 | ENSG00000162607 | 1.14 | 4.06E-02 | Cysteine peptidase |
| ENSMUSG00000029223 | Uchl1 | ENSG00000154277 | 1.16 | 7.50E-03 | Cysteine peptidase |
| ENSMUSG00000050994 | Adgb | ENSG00000118492 | 1.22 | 2.90E-02 | Cysteine peptidase |
| ENSMUSG00000021190 | Lgmn | ENSG00000100600 | 1.22 | 4.86E-06 | Cysteine peptidase |
| ENSMUSG00000019850 | Tnfaip3 | ENSG00000118503 | 1.32 | 7.28E-03 | Cysteine peptidase |
| ENSMUSG00000028776 | Tinagl1 | ENSG00000142910 | 1.34 | 9.20E-03 | Cysteine peptidase |
| ENSMUSG00000050345 | 4930486L24Rik | ENSG00000135047 | 3.01 | 2.15E-02 | Cysteine peptidase |
| ENSMUSG00000008438 | Adam21 | ENSG00000139985 | -1.78 | 3.85E-02 | Metallopeptidase |
| ENSMUSG00000023845 | Lnpep | ENSG00000113441 | -1.67 | 1.96E-05 | Metallopeptidase |
| ENSMUSG00000050663 | Trhde | ENSG00000072657 | -1.64 | 1.82E-10 | Metallopeptidase |
| ENSMUSG00000053399 | Adamts18 | ENSG00000140873 | -1.59 | 9.55E-03 | Metallopeptidase |
| ENSMUSG00000006403 | Adamts4 | ENSG00000158859 | -1.57 | 5.24E-06 | Metallopeptidase |
| ENSMUSG00000073530 | Pappa2 | ENSG00000116183 | -1.55 | 3.79E-03 | Metallopeptidase |
| ENSMUSG00000028226 | Mmp16 | ENSG00000156103 | -1.45 | 2.68E-04 | Metallopeptidase |
| ENSMUSG00000022449 | Adamts20 | ENSG00000173157 | -1.42 | 3.50E-03 | Metallopeptidase |
| ENSMUSG00000040537 | Adam22 | ENSG00000008277 | -1.37 | 2.55E-09 | Metallopeptidase |
| ENSMUSG00000025964 | Adam23 | ENSG00000114948 | -1.36 | 2.12E-08 | Metallopeptidase |
| ENSMUSG00000020926 | Adam11 | ENSG00000073670 | -1.29 | 3.39E-05 | Metallopeptidase |
| ENSMUSG00000011256 | Adam19 | ENSG00000135074 | -1.26 | 5.04E-03 | Metallopeptidase |
| ENSMUSG00000029436 | Mmp17 | ENSG00000198598 | -1.22 | 5.86E-05 | Metallopeptidase |
| ENSMUSG00000024299 | Adamts10 | ENSG00000142303 | -1.22 | 2.72E-02 | Metallopeptidase |
| ENSMUSG00000027612 | Mmp24 | ENSG00000125966 | -1.21 | 2.12E-03 | Metallopeptidase |
| ENSMUSG00000054693 | Adam10 | ENSG00000137845 | -1.15 | 1.13E-02 | Metallopeptidase |
| ENSMUSG00000030884 | Uqcrc2 | ENSG00000140740 | 1.10 | 4.75E-02 | Metallopeptidase |
| ENSMUSG00000029017 | Pmpcb | ENSG00000105819 | 1.12 | 4.74E-02 | Metallopeptidase |
| ENSMUSG00000063931 | Pepd | ENSG00000124299 | 1.13 | 4.97E-02 | Metallopeptidase |
| ENSMUSG00000020681 | Ace | ENSG00000159640 | 1.20 | 1.88E-02 | Metallopeptidase |
| ENSMUSG00000031029 | Eif3f | ENSG00000175390 | 1.23 | 4.69E-05 | Metallopeptidase |
| ENSMUSG00000039062 | Anpep | ENSG00000166825 | 1.24 | 4.93E-03 | Metallopeptidase |
| ENSMUSG00000024644 | Cndp2 | ENSG00000133313 | 1.26 | 8.50E-05 | Metallopeptidase |
| ENSMUSG00000054555 | Adam12 | ENSG00000148848 | 1.27 | 2.76E-02 | Metallopeptidase |
| ENSMUSG00000036545 | Adamts2 | ENSG00000087116 | 1.36 | 1.14E-03 | Metallopeptidase |
| ENSMUSG00000000957 | Mmp14 | ENSG00000157227 | 1.41 | 3.44E-04 | Metallopeptidase |
| ENSMUSG00000029061 | Mmp23 | ENSG00000189409 | 1.52 | 4.02E-03 | Metallopeptidase |
| ENSMUSG00000025355 | Mmp19 | ENSG00000123342 | 1.54 | 2.63E-05 | Metallopeptidase |
| ENSMUSG00000025473 | Adam8 | ENSG00000151651 | 1.56 | 3.03E-03 | Metallopeptidase |
| ENSMUSG00000029718 | Pcolce | ENSG00000106333 | 1.62 | 1.11E-07 | Metallopeptidase |
| ENSMUSG00000070867 | Trabd2b | ENSG00000269113 | 1.62 | 1.78E-05 | Metallopeptidase |
| ENSMUSG00000019278 | Dpep1 | ENSG00000015413 | 1.71 | 2.59E-07 | Metallopeptidase |
| ENSMUSG00000050578 | Mmp13 | ENSG00000137745 | 1.85 | 3.59E-09 | Metallopeptidase |
| ENSMUSG00000031740 | Mmp2 | ENSG00000087245 | 1.88 | 9.13E-05 | Metallopeptidase |
| ENSMUSG00000053687 | Dpep2 | ENSG00000167261 | 1.93 | 3.73E-02 | Metallopeptidase |
| ENSMUSG00000057457 | Phex | ENSG00000102174 | 2.10 | 1.08E-02 | Metallopeptidase |
| ENSMUSG00000024481 | Lvrn | ENSG00000172901 | 2.20 | 3.85E-03 | Metallopeptidase |
| ENSMUSG00000022894 | Adamts5 | ENSG00000154736 | 2.41 | 6.19E-20 | Metallopeptidase |
| ENSMUSG00000017737 | Mmp9 | ENSG00000100985 | 2.55 | 4.54E-18 | Metallopeptidase |
| ENSMUSG00000043613 | Mmp3 | ENSG00000149968 | 3.40 | 5.21E-09 | Metallopeptidase |
| ENSMUSG00000049723 | Mmp12 | ENSG00000262406 | 9.10 | 3.43E-21 | Metallopeptidase |
| ENSMUSG00000025917 | Cops5 | ENSG00000121022 | 1.17 | 6.01E-03 | Metallopeptidase |
| ENSMUSG00000063177 | Klk1b27 |  | -7.46 | 1.42E-03 | Serine peptidase |
| ENSMUSG00000063089 | Klk1b8 |  | -7.26 | 2.11E-03 | Serine peptidase |
| ENSMUSG00000030713 | Klk7 | ENSG00000169035 | -6.89 | 3.68E-02 | Serine peptidase |
| ENSMUSG00000038968 | Klk1b16 |  | -5.78 | 1.85E-03 | Serine peptidase |
| ENSMUSG00000063133 | Klk1b1 | ENSG00000167751 | -5.47 | 1.90E-03 | Serine peptidase |
| ENSMUSG00000066516 | Klk1b21 | ENSG00000167751 | -4.96 | 2.68E-03 | Serine peptidase |
| ENSMUSG00000059042 | Klk1b9 | ENSG00000167751 | -4.96 | 2.09E-03 | Serine peptidase |
| ENSMUSG00000063713 | Klk1b24 | ENSG00000167751 | -4.88 | 3.06E-03 | Serine peptidase |
| ENSMUSG00000066515 | Klk1b3 | ENSG00000167751 | -4.61 | 3.70E-03 | Serine peptidase |
| ENSMUSG00000053719 | Klk1b26 |  | -4.27 | 4.73E-03 | Serine peptidase |
| ENSMUSG00000044485 | Klk1b11 | ENSG00000167751 | -4.26 | 4.65E-03 | Serine peptidase |
| ENSMUSG00000060177 | Klk1b22 | ENSG00000167751 | -4.17 | 6.65E-03 | Serine peptidase |
| ENSMUSG00000066513 | Klk1b4 | ENSG00000167751 | -4.09 | 3.66E-03 | Serine peptidase |
| ENSMUSG00000061780 | Cfd | ENSG00000197766 | -2.40 | 3.07E-31 | Serine peptidase |
| ENSMUSG00000006179 | Prss16 | ENSG00000112812 | -2.16 | 2.71E-02 | Serine peptidase |
| ENSMUSG00000016493 | Cd46 | ENSG00000117335 | -1.52 | 1.26E-03 | Serine peptidase |
| ENSMUSG00000070695 | Cntnap5a | ENSG00000155052 | -1.51 | 4.74E-07 | Serine peptidase |
| ENSMUSG00000042453 | Reln | ENSG00000189056 | -1.42 | 2.02E-07 | Serine peptidase |
| ENSMUSG00000037129 | Tmprss13 | ENSG00000137747 | -1.38 | 2.38E-02 | Serine peptidase |
| ENSMUSG00000028979 | Masp2 | ENSG00000009724 | -1.35 | 5.29E-05 | Serine peptidase |
| ENSMUSG00000032393 | Dpp8 | ENSG00000074603 | -1.29 | 2.28E-05 | Serine peptidase |
| ENSMUSG00000031995 | St14 | ENSG00000149418 | -1.29 | 3.87E-02 | Serine peptidase |
| ENSMUSG00000036098 | Myrf | ENSG00000124920 | -1.27 | 2.90E-04 | Serine peptidase |
| ENSMUSG00000021587 | Pcsk1 | ENSG00000175426 | -1.21 | 4.27E-02 | Serine peptidase |
| ENSMUSG00000001229 | Dpp9 | ENSG00000142002 | -1.20 | 1.20E-03 | Serine peptidase |
| ENSMUSG00000024127 | Prepl | ENSG00000138078 | -1.19 | 3.01E-03 | Serine peptidase |
| ENSMUSG00000061576 | Dpp6 | ENSG00000130226 | -1.16 | 9.53E-03 | Serine peptidase |
| ENSMUSG00000025246 | Tbl1x | ENSG00000092377 | -1.13 | 2.86E-02 | Serine peptidase |
| ENSMUSG00000047866 | Lonp2 | ENSG00000102910 | 1.14 | 6.23E-03 | Serine peptidase |
| ENSMUSG00000000278 | Scpep1 | ENSG00000121064 | 1.17 | 3.49E-02 | Serine peptidase |
| ENSMUSG00000006205 | Htra1 | ENSG00000166033 | 1.17 | 2.64E-02 | Serine peptidase |
| ENSMUSG00000017760 | Ctsa | ENSG00000064601 | 1.18 | 5.18E-04 | serine peptidase |
| ENSMUSG00000024713 | Pcsk5 | ENSG00000099139 | 1.22 | 1.61E-03 | Serine peptidase |
| ENSMUSG00000021822 | Plau | ENSG00000122861 | 1.25 | 3.64E-02 | Serine peptidase |
| ENSMUSG00000027188 | Pamr1 | ENSG00000149090 | 1.27 | 3.85E-03 | Serine peptidase |
| ENSMUSG00000020323 | Prss57 | ENSG00000185198 | 1.46 | 1.85E-02 | Serine peptidase |
| ENSMUSG00000033491 | Prss35 | ENSG00000146250 | 1.51 | 1.57E-03 | Serine peptidase |
| ENSMUSG00000000392 | Fap | ENSG00000078098 | 1.54 | 1.07E-06 | Serine peptidase |
| ENSMUSG00000055172 | C1ra | ENSG00000159403 | 1.59 | 2.50E-05 | Serine peptidase |
| ENSMUSG00000098470 | C1rb | ENSG00000159403 | 1.70 | 1.63E-04 | Serine peptidase |
| ENSMUSG00000038521 | C1s1 | ENSG00000182326 | 1.70 | 3.05E-09 | Serine peptidase |
| ENSMUSG00000090231 | Cfb | ENSG00000243649 | 2.31 | 1.94E-23 | Serine peptidase |
| ENSMUSG00000029096 | Htra3 | ENSG00000170801 | 2.54 | 3.35E-30 | Serine peptidase |
| ENSMUSG00000021492 | F12 | ENSG00000131187 | 2.63 | 2.46E-02 | Serine peptidase |
| ENSMUSG00000041534 | Rbp3 | ENSG00000265203 | 2.76 | 2.92E-04 | Serine peptidase |
| ENSMUSG00000061068 | Mcpt4 |  | 3.01 | 1.67E-22 | Serine peptidase |
| ENSMUSG00000033825 | Tpsb2 | ENSG00000095917, ENSG00000172236, ENSG00000197253 | 3.02 | 1.72E-18 | Serine peptidase |
| ENSMUSG00000023031 | Cela1 | ENSG00000139610 | 3.48 | 8.79E-11 | Serine peptidase |
| ENSMUSG00000022225 | Cma1 | ENSG00000092009 | 3.85 | 2.95E-20 | Serine peptidase |
| ENSMUSG00000049719 | Prss46 | ENSG00000261603 | 4.82 | 1.12E-02 | Serine peptidase |
| ENSMUSG00000031443 | F7 | ENSG00000057593 | 5.27 | 1.33E-02 | Serine peptidase |
| ENSMUSG00000031722 | Hp | ENSG00000261701 | 7.95 | 1.62E-147 | Serine peptidase |
| ENSMUSG00000026750 | Psmb7 | ENSG00000136930 | 1.12 | 1.50E-02 | Threonine peptidase |
| ENSMUSG00000030751 | Psma1 | ENSG00000256206 | 1.12 | 4.82E-02 | Threonine peptidase |
| ENSMUSG00000068749 | Psma5 | ENSG00000143106 | 1.15 | 4.69E-02 | Threonine peptidase |
| ENSMUSG00000030591 | Psmd8 | ENSG00000099341 | 1.16 | 2.00E-03 | Threonine peptidase |
| ENSMUSG00000042541 | Shfm1 |  | 1.16 | 3.58E-02 | Threonine peptidase |
| ENSMUSG00000022193 | Psmb5 | ENSG00000100804 | 1.16 | 1.74E-02 | Threonine peptidase |
| ENSMUSG00000015671 | Psma2 | ENSG00000256646 | 1.18 | 1.23E-03 | Threonine peptidase |
| ENSMUSG00000027566 | Psma7 | ENSG00000101182 | 1.19 | 3.32E-04 | Threonine peptidase |
| ENSMUSG00000039033 | Tasp1 | ENSG00000089123 | 1.20 | 2.12E-02 | Threonine peptidase |
| ENSMUSG00000005779 | Psmb4 | ENSG00000159377 | 1.20 | 1.58E-04 | Threonine peptidase |
| ENSMUSG00000014769 | Psmb1 | ENSG00000008018 | 1.20 | 3.07E-04 | Threonine peptidase |
| ENSMUSG00000031897 | Psmb10 | ENSG00000205220 | 1.21 | 1.07E-02 | Threonine peptidase |
| ENSMUSG00000021024 | Psma6 | ENSG00000100902 | 1.21 | 7.23E-04 | Threonine peptidase |
| ENSMUSG00000024338 | Psmb8 | ENSG00000204264 | 1.22 | 4.43E-02 | Threonine peptidase |
| ENSMUSG00000018286 | Psmb6 | ENSG00000142507 | 1.24 | 8.80E-05 | Threonine peptidase |
| ENSMUSG00000028837 | Psmb2 | ENSG00000126067 | 1.30 | 6.18E-06 | Threonine peptidase |
| ENSMUSG00000006344 | Ggt5 | ENSG00000099998 | 1.59 | 2.00E-03 | Threonine peptidase |

**Table S3: Gene lists and classification of GO term proteolysis from transcriptomes of mice head.**

**Table S4**

| **Zebrafish ID** | **Zebrafish gene name** | **Human homolog** | **Meas/Ctrl or -Ctrl/Meas (scaled)** | **p-value** | **Classification** |
| --- | --- | --- | --- | --- | --- |
| ENSDARG00000057698 | ctsd | ENSG00000117984 | 1.15 | 3.20E-02 | Aspartic peptidase |
| ENSDARG00000008165 | caspa |  | -2.08 | 4.23E-03 | Cysteine protease |
| ENSDARG00000052039 | caspb |  | -2.04 | 1.50E-05 | Cysteine protease |
| ENSDARG00000052917 | si:ch211-202f3.3 | ENSG00000214711 | -1.84 | 1.15E-03 | Cysteine protease |
| ENSDARG00000005595 | adgb | ENSG00000118492 | -1.75 | 2.08E-02 | Cysteine protease |
| ENSDARG00000013771 | ctss2.2 | ENSG00000163131 | -1.68 | 2.27E-02 | Cysteine protease |
| ENSDARG00000034211 | capn2l | ENSG00000162909 | -1.68 | 3.87E-04 | Cysteine protease |
| ENSDARG00000012341 | capn9 | ENSG00000135773 | -1.54 | 7.25E-03 | Cysteine protease |
| ENSDARG00000045641 | usp3 | ENSG00000140455 | -1.49 | 1.75E-02 | Cysteine protease |
| ENSDARG00000098239 | zgc:85932 |  | -1.46 | 2.26E-03 | Cysteine protease |
| ENSDARG00000091699 | capn2a |  | -1.42 | 1.87E-03 | Cysteine protease |
| ENSDARG00000030177 | uchl3 | ENSG00000118939 | -1.40 | 2.22E-03 | Cysteine protease |
| ENSDARG00000055045 | casp3b | ENSG00000164305 | -1.37 | 2.73E-02 | Cysteine protease |
| ENSDARG00000040990 | usp37 | ENSG00000135913 | -1.31 | 6.36E-03 | Cysteine protease |
| ENSDARG00000035329 | capns1a | ENSG00000126247 | -1.31 | 2.15E-02 | Cysteine protease |
| ENSDARG00000013804 | capns1b | ENSG00000126247 | -1.23 | 2.72E-02 | Cysteine protease |
| ENSDARG00000089861 | usp44 | ENSG00000136014 | 1.19 | 4.32E-02 | Cysteine protease |
| ENSDARG00000063190 | zranb1b | ENSG00000019995 | 1.19 | 2.87E-02 | Cysteine protease |
| ENSDARG00000079198 | usp13 | ENSG00000058056 | 1.28 | 1.63E-02 | Cysteine protease |
| ENSDARG00000102705 | otud6b | ENSG00000155100 | 1.30 | 2.81E-02 | Cysteine protease |
| ENSDARG00000019595 | senp8 | ENSG00000166192 | 1.82 | 3.25E-02 | Cysteine protease |
| ENSDARG00000101051 | ctsbb | ENSG00000136943 | 1.92 | 4.83E-05 | Cysteine protease |
| ENSDARG00000052578 | c6ast4 |  | 1.97 | 1.26E-08 | Cysteine protease |
| ENSDARG00000069748 | capn5b | ENSG00000149260 | 2.02 | 4.80E-02 | Cysteine protease |
| ENSDARG00000088145 | atg4db | ENSG00000130734 | 4.19 | 1.55E-02 | Cysteine protease |
| ENSDARG00000042816 | mmp9 | ENSG00000100985 | -3.26 | 5.48E-03 | Metallopeptidase |
| ENSDARG00000059029 | mmp28 | ENSG00000271447 | -2.30 | 2.67E-02 | Metallopeptidase |
| ENSDARG00000045887 | mmp30 |  | -1.66 | 4.76E-08 | Metallopeptidase |
| ENSDARG00000034693 | mysm1 | ENSG00000162601 | 1.23 | 3.98E-02 | Metallopeptidase |
| ENSDARG00000067545 | adam19b | ENSG00000135074 | 1.24 | 3.16E-02 | Metallopeptidase |
| ENSDARG00000062363 | phex | ENSG00000102174 | 1.24 | 4.58E-02 | Metallopeptidase |
| ENSDARG00000068187 | spg7 | ENSG00000197912 | 1.25 | 4.21E-02 | Metallopeptidase |
| ENSDARG00000079166 | ace | ENSG00000264813 | 1.32 | 1.78E-02 | Metallopeptidase |
| ENSDARG00000007813 | rnpepl1 | ENSG00000142327 | 1.36 | 1.19E-02 | Metallopeptidase |
| ENSDARG00000006901 | si:ch1073-459j12.1 | ENSG00000106624 | 1.40 | 5.42E-03 | Metallopeptidase |
| ENSDARG00000061737 | ece1 | ENSG00000117298 | 1.52 | 1.38E-04 | Metallopeptidase |
| ENSDARG00000043722 | cpa4 | ENSG00000158516 | 1.60 | 2.81E-03 | Metallopeptidase |
| ENSDARG00000006029 | lta4h | ENSG00000111144 | 1.88 | 3.17E-04 | Metallopeptidase |
| ENSDARG00000057644 | adam8b | ENSG00000151651 | 2.05 | 4.07E-04 | Metallopeptidase |
| ENSDARG00000079983 | agbl2 | ENSG00000165923 | 4.57 | 2.56E-03 | Metallopeptidase |
| ENSDARG00000059026 | zgc:123217 | ENSG00000189099 | -6.64 | 3.52E-02 | Serine peptidase |
| ENSDARG00000077540 | f2rl1.2 | ENSG00000164251 | -1.98 | 2.11E-02 | Serine peptidase |
| ENSDARG00000039579 | cfd | ENSG00000197766 | -1.90 | 2.20E-04 | Serine peptidase |
| ENSDARG00000038891 | AL954146.1 |  | -1.86 | 4.30E-02 | Serine peptidase |
| ENSDARG00000079393 | tmprss15 |  | -1.79 | 1.10E-02 | Serine peptidase |
| ENSDARG00000055014 | si:dkey-33m11.8 |  | -1.65 | 1.46E-02 | Serine peptidase |
| ENSDARG00000004748 | zgc:100868 | ENSG00000103355 | -1.65 | 1.62E-05 | Serine peptidase |
| ENSDARG00000095807 | hp | ENSG00000263639 | -1.59 | 1.56E-02 | Serine peptidase |
| ENSDARG00000032831 | htra1a | ENSG00000166033 | -1.52 | 1.56E-02 | Serine peptidase |
| ENSDARG00000102332 | spint1a | ENSG00000243543 | -1.49 | 6.25E-04 | Serine peptidase |
| ENSDARG00000058593 | sri | ENSG00000075142 | -1.38 | 3.03E-03 | Serine peptidase |
| ENSDARG00000089138 | si:ch1073-440b2.1 | ENSG00000170500 | -1.32 | 4.82E-03 | Serine peptidase |
| ENSDARG00000061173 | st14a |  | -1.32 | 1.80E-02 | Serine peptidase |
| ENSDARG00000100691 | prss35 | ENSG00000146250 | -1.30 | 2.69E-02 | Serine peptidase |
| ENSDARG00000037783 | proza | ENSG00000126231 | 1.15 | 3.49E-02 | Serine peptidase |
| ENSDARG00000029063 | clpxa | ENSG00000166855 | 1.23 | 3.80E-04 | Serine peptidase |
| ENSDARG00000088581 | f10 | ENSG00000126218 | 1.24 | 1.31E-02 | Serine peptidase |
| ENSDARG00000075048 | lonrf1 |  | 1.29 | 4.25E-03 | Serine peptidase |
| ENSDARG00000037883 | prcp | ENSG00000137509 | 1.45 | 2.48E-02 | Serine peptidase |
| ENSDARG00000073742 | prss59.2 |  | 1.62 | 1.27E-02 | Serine peptidase |
| ENSDARG00000045544 | hgfa | ENSG00000019991 | 1.65 | 1.46E-02 | Serine peptidase |
| ENSDARG00000078567 | lonrf1l | ENSG00000154359 | 1.67 | 2.69E-06 | Serine peptidase |
| ENSDARG00000093844 | zgc:136461 | ENSG00000168928 | 1.78 | 6.57E-11 | Serine peptidase |
| ENSDARG00000056765 | ela2l | ENSG00000142615 | 1.82 | 2.41E-12 | Serine peptidase |
| ENSDARG00000042993 | prss1 | ENSG00000204983 | 1.84 | 1.10E-10 | Serine peptidase |
| ENSDARG00000068680 | ctrl | ENSG00000141086 | 1.89 | 1.72E-09 | Serine peptidase |
| ENSDARG00000007276 | ela3l |  | 1.90 | 1.12E-09 | Serine peptidase |
| ENSDARG00000079274 | prss59.1 |  | 1.95 | 1.41E-09 | Serine peptidase |
| ENSDARG00000056744 | ela2 | ENSG00000142615 | 2.03 | 2.05E-02 | Serine peptidase |
| ENSDARG00000090428 | ctrb1 | ENSG00000168925 | 2.19 | 8.72E-20 | Serine peptidase |
| ENSDARG00000094741 | HTRA2 (1 of many) | ENSG00000115317 | 2.21 | 4.19E-02 | Serine peptidase |
| ENSDARG00000017314 | CELA1 (1 of many) | ENSG00000139610 | 2.27 | 5.10E-16 | Serine peptidase |
| ENSDARG00000043173 | CELA1 (1 of many) | ENSG00000139610 | 3.87 | 6.95E-03 | Serine peptidase |
| ENSDARG00000002240 | psmb6 | ENSG00000142507 | 1.39 | 7.79E-03 | Threonine peptidase |
| ENSDARG00000043781 | psmb10 | ENSG00000205220 | 1.93 | 5.73E-03 | Threonine peptidase |

**Table S4: Gene lists and classification of GO term proteolysis from transcriptomes of zebrafish larvae.**

**Table S5**

| **Mouse ID** | **Mouse gene_name** | **Human gene stable ID** | **Meas/Ctrl or -Ctrl/Meas (scaled)** | **p-value** | **p-adj** |
| --- | --- | --- | --- | --- | --- |
| ENSMUSG00000025479 | Cyp2e1 | ENSG00000130649 | -3.45 | 2.60E-31 | 3.66E-28 |
| ENSMUSG00000060675 | Pla2g16 | ENSG00000176485 | 1.4 | 3.29E-11 | 4.28E-09 |
| ENSMUSG00000028597 | Gpx7 | ENSG00000116157 | 1.85 | 3.54E-07 | 1.55E-05 |
| ENSMUSG00000015090 | Ptgds | ENSG00000107317 | 1.25 | 6.88E-07 | 2.78E-05 |
| ENSMUSG00000020377 | Ltc4s | ENSG00000213316 | 2.45 | 1.74E-06 | 6.17E-05 |
| ENSMUSG00000017969 | Ptgis | ENSG00000124212 | 1.43 | 3.72E-05 | 7.88E-04 |
| ENSMUSG00000041193 | Pla2g5 | ENSG00000127472 | 1.67 | 7.24E-05 | 1.36E-03 |
| ENSMUSG00000018339 | Gpx3 | ENSG00000211445 | 1.27 | 2.94E-04 | 4.25E-03 |
| ENSMUSG00000063856 | Gpx1 | ENSG00000233276 | 1.28 | 7.91E-04 | 9.20E-03 |
| ENSMUSG00000034579 | Pla2g3 | ENSG00000100078 | -1.26 | 1.34E-03 | 1.41E-02 |
| ENSMUSG00000021760 | Gpx8 | ENSG00000164294 | 1.31 | 1.92E-03 | 1.86E-02 |
| ENSMUSG00000006344 | Ggt5 | ENSG00000099998 | 1.59 | 2.00E-03 | 1.92E-02 |
| ENSMUSG00000022040 | Ephx2 | ENSG00000120915 | 1.27 | 2.47E-03 | 2.24E-02 |
| ENSMUSG00000098488 | Pla2g4b | ENSG00000168970 | -1.72 | 2.50E-03 | 2.26E-02 |
| ENSMUSG00000022947 | Cbr3 | ENSG00000159231 | 1.31 | 9.26E-03 | 5.94E-02 |
| ENSMUSG00000029059 | Fam213b | ENSG00000157870 | 1.18 | 1.12E-02 | 6.79E-02 |
| ENSMUSG00000027999 | Pla2g12a | ENSG00000123739 | 1.19 | 1.48E-02 | 8.27E-02 |
| ENSMUSG00000042808 | Gpx2 | ENSG00000176153 | 1.33 | 2.20E-02 | 1.09E-01 |
| ENSMUSG00000056220 | Pla2g4a | ENSG00000116711 | 1.33 | 2.20E-02 | 1.09E-01 |
| ENSMUSG00000020891 | Alox8 | ENSG00000179593 | -1.5 | 2.31E-02 | 1.13E-01 |
| ENSMUSG00000052914 | Cyp2j6 | ENSG00000134716 | 1.16 | 3.04E-02 | 1.36E-01 |

**Table S5: Gene lists of GO term arachidonic acid metabolism from transcriptomes of mice head.**

**Table S6**

| **Mouse ID** | **Mouse gene name** | **Human gene stable ID** | **MEAS/CTRL or -CTRL/MEAS (scaled)** | **p value** | **q value** |
| --- | --- | --- | --- | --- | --- |
| ENSMUSG00000006344 | Ggt5 | ENSG00000099998 | 1.56 | 2.14E-03 | 2.60E-03 |
| ENSMUSG00000009646 | Pla2g12b | ENSG00000138308 | 1.57 | 1.35E-03 | 1.74E-03 |
| ENSMUSG00000018339 | Gpx3 | ENSG00000211445 | 2.56 | 4.80E-16 | 4.35E-15 |
| ENSMUSG00000022040 | Ephx2 | ENSG00000120915 | -1.57 | 1.49E-11 | 8.59E-11 |
| ENSMUSG00000022947 | Cbr3 | ENSG00000159231 | 13.91 | 2.33E-31 | 5.53E-30 |
| ENSMUSG00000024055 | Cyp4f13 | ENSG00000186526 | -1.51 | 2.12E-14 | 1.65E-13 |
| ENSMUSG00000024292 | Cyp4f14 | ENSG00000186115 | -1.73 | 2.32E-12 | 1.48E-11 |
| ENSMUSG00000025002 | Cyp2c55 | ENSG00000108242 | 4.51 | 2.08E-24 | 3.47E-23 |
| ENSMUSG00000025003 | Cyp2c39 |  | 3.16 | 5.14E-31 | 1.19E-29 |
| ENSMUSG00000025004 | Cyp2c40 |  | 1.78 | 8.28E-03 | 8.27E-03 |
| ENSMUSG00000025197 | Cyp2c23 |  | -3.84 | 1.62E-55 | 1.17E-53 |
| ENSMUSG00000025479 | Cyp2e1 | ENSG00000130649 | -1.48 | 5.25E-10 | 2.51E-09 |
| ENSMUSG00000026820 | Ptges2 | ENSG00000148334 | 1.34 | 4.51E-03 | 4.95E-03 |
| ENSMUSG00000027983 | Cyp2u1 | ENSG00000155016 | -2.61 | 1.72E-14 | 1.36E-13 |
| ENSMUSG00000028597 | Gpx7 | ENSG00000116157 | 2.48 | 3.39E-06 | 8.52E-06 |
| ENSMUSG00000028712 | Cyp4a31 | ENSG00000186204 | -4.29 | 1.03E-25 | 1.83E-24 |
| ENSMUSG00000028715 | Cyp4a14 |  | -2.02 | 1.69E-20 | 2.21E-19 |
| ENSMUSG00000029919 | Hpgds | ENSG00000163106 | 3.37 | 1.28E-09 | 5.76E-09 |
| ENSMUSG00000029925 | Tbxas1 | ENSG00000059377 | 2.32 | 5.19E-06 | 1.26E-05 |
| ENSMUSG00000030483 | Cyp2b10 |  | -1.33 | 4.18E-02 | 3.20E-02 |
| ENSMUSG00000032808 | Cyp2c38 |  | 1.82 | 6.04E-10 | 2.86E-09 |
| ENSMUSG00000040660 | Cyp2b9 |  | 79.13 | 1.13E-46 | 5.73E-45 |
| ENSMUSG00000042248 | Cyp2c37 |  | -3.39 | 1.76E-47 | 9.41E-46 |
| ENSMUSG00000042632 | Pla2g6 | ENSG00000184381 | 1.95 | 1.23E-07 | 4.06E-07 |
| ENSMUSG00000047250 | Ptgs1 | ENSG00000095303 | 1.34 | 3.18E-03 | 3.66E-03 |
| ENSMUSG00000051483 | Cbr1 | ENSG00000159228 | 2.26 | 4.09E-10 | 1.98E-09 |
| ENSMUSG00000052520 | Cyp2j5 |  | -1.14 | 3.47E-03 | 3.96E-03 |
| ENSMUSG00000054827 | Cyp2c50 |  | -1.74 | 1.71E-09 | 7.60E-09 |
| ENSMUSG00000056220 | Pla2g4a | ENSG00000116711 | 1.24 | 2.08E-03 | 2.54E-03 |
| ENSMUSG00000063856 | Gpx1 | ENSG00000233276 | 1.48 | 4.96E-03 | 5.35E-03 |
| ENSMUSG00000063929 | Cyp4a32 | ENSG00000186204 | -1.95 | 2.11E-19 | 2.56E-18 |
| ENSMUSG00000066072 | Cyp4a10 | ENSG00000186204 | -2.10 | 3.56E-16 | 3.31E-15 |
| ENSMUSG00000067225 | Cyp2c54 |  | -4.10 | 8.56E-34 | 2.24E-32 |
| ENSMUSG00000071072 | Ptges3 | ENSG00000110958 | -1.21 | 3.96E-04 | 5.99E-04 |
| ENSMUSG00000074882 | Cyp2c68 |  | 1.54 | 1.35E-05 | 3.00E-05 |
| ENSMUSG00000078597 | Cyp4a12b |  | -5.43 | 2.94E-17 | 3.02E-16 |

**Table S6: Gene lists of GO term arachidonic acid metabolism from transcriptomes of published mice liver.**

**Table S7**

| **Zebrafish ID** | **Fish gene_name** | **Human gene stable ID** | **Meas/Ctrl or -Ctrl/Meas (scaled)** | **p-value** | **p-adj** |
| --- | --- | --- | --- | --- | --- |
| ENSDARG00000060094 | ptgis | ENSG00000124212 | -2.21 | 4.57E-02 | 4.37E-01 |
| ENSDARG00000004539 | ptgs2a | ENSG00000073756 | -1.66 | 1.21E-03 | 5.49E-02 |
| ENSDARG00000010276 | ptgs2b | ENSG00000073756 | -1.60 | 2.86E-03 | 9.78E-02 |
| ENSDARG00000027088 | ptgdsb.1 |  | -1.52 | 7.38E-05 | 7.29E-03 |
| ENSDARG00000021149 | cbr1l |  | -1.48 | 8.79E-04 | 4.48E-02 |
| ENSDARG00000069463 | alox12 | ENSG00000108839 | -1.27 | 4.13E-02 | 4.19E-01 |
| ENSDARG00000089626 | ptges3b | ENSG00000110958 | -1.24 | 2.18E-02 | 3.07E-01 |
| ENSDARG00000006029 | lta4h | ENSG00000111144 | 1.88 | 3.17E-04 | 2.16E-02 |
| ENSDARG00000009153 | pla2g1b |  | 2.15 | 4.19E-02 | 4.22E-01 |
| ENSDARG00000042090 | si:ch73-55i23.1 | ENSG00000105499 | 2.81 | 3.51E-02 | 3.88E-01 |

**Table S7: Gene lists of GO term arachidonic acid metabolism from transcriptomes of zebrafish larvae.**

**Table S8**

| Human homologs | Human gene name | Mice id | Mice head MEAS/CTRL or -CTRL/MEAS (scaled) | Mice head p-value | Mice liver MEAS/CTRL or -CTRL/MEAS (scaled) | Mice liver p-value | Fish ID | Fish MEAS/CTRL or -CTRL/MEAS (scaled) | Fish p-value |
| --- | --- | --- | --- | --- | --- | --- | --- | --- | --- |
| ENSG00000127472 | PLA2G5 | ENSMUSG00000041193 | 1.67 | 7.24E-05 |  |  |  |  |  |
| ENSG00000116711 | PLA2G4A | ENSMUSG00000056220 | 1.33 | 2.20E-02 | 1.24 | 2.08E-03 |  |  |  |
| ENSG00000179593 | ALOX15B | ENSMUSG00000020891 | -1.50 | 2.31E-02 |  |  |  |  |  |
| ENSG00000132965 | ALOX5AP | ENSMUSG00000060063 | 1.74 | 1.81E-06 | 1.76 | 1.04E-02 |  |  |  |
| ENSG00000213316 | LTC4S | ENSMUSG00000020377 | 2.45 | 1.74E-06 |  |  |  |  |  |
| ENSG00000015413 | DPEP1 | ENSMUSG00000019278 | 1.71 | 2.59E-07 | 1.59 | 5.63E-03 |  |  |  |
| ENSG00000107317 | PTGDS | ENSMUSG00000015090 | 1.25 | 6.88E-07 |  |  |  |  |  |
| ENSG00000151726 | ACSL1 | ENSMUSG00000018796 | 1.31 | 2.80E-06 | -1.34 | 2.65E-05 |  |  |  |
| ENSG00000182718 | ANXA2 | ENSMUSG00000032231 | 1.63 | 2.56E-13 | 10.01 | 3.36E-90 |  |  |  |
| ENSG00000138772 | ANXA3 | ENSMUSG00000029484 | 1.48 | 1.82E-06 | 1.82 | 3.72E-06 |  |  |  |
| ENSG00000196975 | ANXA4 | ENSMUSG00000029994 | 1.33 | 1.24E-04 | 1.40 | 1.80E-04 |  |  |  |
| ENSG00000197043 | ANXA6 | ENSMUSG00000018340 | 1.10 | 4.38E-02 |  |  |  |  |  |
| ENSG00000123983 | ACSL3 | ENSMUSG00000032883 |  |  | 1.42 | 4.74E-14 | ENSDARG00000032079 | 2.23 | 2.61E-02 |
| ENSG00000073756 | PTGS2 |  |  |  |  |  | ENSDARG00000004539 | -1.66 | 1.21E-03 |
| ENSG00000073756 | PTGS2 |  |  |  |  |  | ENSDARG00000010276 | -1.60 | 2.86E-03 |
| ENSG00000100344 | PNPLA3 |  |  |  |  |  | ENSDARG00000102020 | 1.58 | 6.74E-05 |
| ENSG00000111144 | LTA4H |  |  |  |  |  | ENSDARG00000006029 | 1.88 | 3.17E-04 |
| ENSG00000108839 | ALOX12 |  |  |  |  |  | ENSDARG00000069463 | -1.27 | 4.13E-02 |
| ENSG00000197747 | S100A10 | ENSMUSG00000041959 | 1.44 | 8.09E-11 | 2.65 | 2.06E-12 | ENSDARG00000055589 | -1.69 | 8.71E-05 |
| ENSG00000134716 | CYP2J2 | ENSMUSG00000052914 | 1.16 | 3.04E-02 |  |  | ENSDARG00000098803 | -5.70 | 2.52E-03 |
| ENSG00000135046 | ANXA1 | ENSMUSG00000024659 | 1.67 | 6.86E-08 |  |  | ENSDARG00000100095 | -3.40 | 4.54E-08 |
| ENSG00000135046 | ANXA1 | ENSMUSG00000024659 | 1.67 | 6.86E-08 | 2.32 | 5.94E-08 | ENSDARG00000026726 | -2.57 | 6.82E-03 |
| ENSG00000116044 | NFE2L2 | ENSMUSG00000015839 | 1.15 | 1.72E-02 | 1.65 | 4.46E-06 | ENSDARG00000042824 | -2.25 | 3.39E-02 |
| ENSG00000124212 | PTGIS | ENSMUSG00000017969 | 1.43 | 3.72E-05 |  |  | ENSDARG00000060094 | -2.21 | 4.57E-02 |
| ENSG00000164111 | ANXA5 | ENSMUSG00000027712 | 1.24 | 5.46E-05 | 3.60 | 4.04E-54 | ENSDARG00000026406 | -1.65 | 1.03E-02 |
| ENSG00000108984 | MAP2K6 | ENSMUSG00000020623 | -1.29 | 4.66E-04 | -1.62 | 2.28E-04 | ENSDARG00000099184 | -1.20 | 1.28E-02 |

**Table S8: Gene signature sets of mice head, mice liver and zebrafish larvae in Figure 8.**
